# Anoxygenic photo- and chemo-synthesis of phototrophic sulfur bacteria from an alpine meromictic lake

**DOI:** 10.1093/femsec/fiab010

**Published:** 2021-01-29

**Authors:** Francesco Di Nezio, Clarisse Beney, Samuele Roman, Francesco Danza, Antoine Buetti-Dinh, Mauro Tonolla, Nicola Storelli

**Affiliations:** Laboratory of Applied Microbiology (LMA), Department of Environmental Constructions and Design (DACD), University of Applied Sciences and Arts of Southern Switzerland (SUPSI), via Mirasole 22a, 6500 Bellinzona, Switzerland; Microbiology Unit, Department of Botany and Plant Biology (BIVEG), University of Geneva, Quai Ernest-Ansermet 30, 1211 Geneva 4, 1211 Geneva, Switzerland; Laboratory of Applied Microbiology (LMA), Department of Environmental Constructions and Design (DACD), University of Applied Sciences and Arts of Southern Switzerland (SUPSI), via Mirasole 22a, 6500 Bellinzona, Switzerland; Microbiology Unit, Department of Botany and Plant Biology (BIVEG), University of Geneva, Quai Ernest-Ansermet 30, 1211 Geneva 4, 1211 Geneva, Switzerland; Laboratory of Applied Microbiology (LMA), Department of Environmental Constructions and Design (DACD), University of Applied Sciences and Arts of Southern Switzerland (SUPSI), via Mirasole 22a, 6500 Bellinzona, Switzerland; Alpine Biology Center Foundation, via Mirasole 22a, 6500 Bellinzona, Switzerland; Laboratory of Applied Microbiology (LMA), Department of Environmental Constructions and Design (DACD), University of Applied Sciences and Arts of Southern Switzerland (SUPSI), via Mirasole 22a, 6500 Bellinzona, Switzerland; Laboratory of Applied Microbiology (LMA), Department of Environmental Constructions and Design (DACD), University of Applied Sciences and Arts of Southern Switzerland (SUPSI), via Mirasole 22a, 6500 Bellinzona, Switzerland; Laboratory of Applied Microbiology (LMA), Department of Environmental Constructions and Design (DACD), University of Applied Sciences and Arts of Southern Switzerland (SUPSI), via Mirasole 22a, 6500 Bellinzona, Switzerland; Microbiology Unit, Department of Botany and Plant Biology (BIVEG), University of Geneva, Quai Ernest-Ansermet 30, 1211 Geneva 4, 1211 Geneva, Switzerland; Alpine Biology Center Foundation, via Mirasole 22a, 6500 Bellinzona, Switzerland; Laboratory of Applied Microbiology (LMA), Department of Environmental Constructions and Design (DACD), University of Applied Sciences and Arts of Southern Switzerland (SUPSI), via Mirasole 22a, 6500 Bellinzona, Switzerland

**Keywords:** chemocline, phototrophic sulfur bacteria, CO_2_ fixation, anoxygenic photosynthesis, chemotrophy

## Abstract

Meromictic lakes are interesting ecosystems to study anaerobic microorganisms due their permanent stratification allowing the formation of a stable anoxic environment. The crenogenic meromictic Lake Cadagno harbors an important community of anoxygenic phototrophic sulfur bacteria responsible for almost half of its total productivity. Besides their ability to fix CO_2_ through photosynthesis, these microorganisms also showed high rates of dark carbon fixation via chemosyntesis. Here, we grew in pure cultures three populations of anoxygenic phototrophic sulfur bacteria previously isolated from the lake, accounting for 72.8% of the total microbial community and exibiting different phenotypes: (1) the motile, large-celled purple sulfur bacterium (PSB) *Chromatium okenii*, (2) the small-celled PSB *Thiodictyon syntrophicum* and (3) the green sulfur bacterium (GSB) *Chlorobium phaeobacteroides*. We measured their ability to fix CO_2_ through photo- and chemo-synthesis, both *in situ* in the lake and in laboratory under different incubation conditions. We also evaluated the efficiency and velocity of H_2_S photo-oxidation, an important reaction in the anoxygenic photosynthesis process. Our results confirm that phototrophic sulfur bacteria strongly fix CO_2_ in the presence of light and that oxygen increases chemosynthesis at night, in laboratory conditions. Moreover, substancial differences were displayed between the three selected populations in terms of activity and abundance.

## Teaser

The ecological role of three different species of anoxygenic phototrophic sulfur bacteria from the chemocline of an alpine meromictic lake was investigated through a combination of field and laboratory experiments.

## INTRODUCTION

Primary production is the conversion of energy and inorganic carbon into organic matter by autotrophic organisms. The most familiar form of primary production is photosynthesis, where carbon dioxide is converted into organic substances drawing energy from sunlight. Not all primary production, however, results from photosynthesis; under anaerobic conditions, some microorganisms are capable of chemosynthetic growth (Yang *et al*. 2011; Smith 2012). Unlike photosynthesis, microbial production by chemosynthesis converts inorganic carbon into organic matter using chemical energy from the oxidation of reduced compounds. Among such microrganisms are anoxygenic phototrophic sulfur bacteria which possess both photo- and chemo-synthetic abilities (Overmann 2008). Anoxygenic phototrophic sulfur bacteria play an important role in anoxic environments where they are the main primary producers organisms fixing inorganic carbon (Frigaard and Dahl 2008). They can photo-oxidize different electron donors such as hydrogen (H_2_), hydrogen sulfide (HS^−^), thiosulfate (S_2_O_3_^2−^), sulfur (S^0^) and reduced iron (Fe^3+^) and they thrive in aquatic environments where both light and sulfide are present, including sediments (Preisler *et al*. 2007), hot springs (Hanada 2003) and freshwater lakes (Sorokin, Kuenen and Muyzer 2011; Watanabe *et al*. 2013).

Meromictic lakes, characterized by a permanent stratification of the water column which allows the formation of a stable anoxic compartment, represent a suitable environment to sustain the growth of anoxygenic phototrophic sulfur bacteria (Zadereev, Gulati and Camacho 2017). Alpine meromictic Lake Cadagno, located in the Ticino canton of Switzerland, has been intensely investigated over the last decades (Tonolla *et al*. 2017). Its chemocline, typically lying at about 12 m depth, separates the oxic, salt-poor surface water of low density from the anoxic, salt-rich bottom water of high density. The opposite gradients of sulfide and light irradiance present in the chemocline support the growth of a dense community of purple sulfur bacteria (PSB) of the genera *Chromatium*, *Lamprocystis*, *Thiocystis* and *Thiodictyon* and green sulfur bacteria (GSB) of the genus *Chlorobium* (Tonolla, Demarta and Peduzzi 1999; Tonolla *et al*. 2003; Tonolla, Peduzzi and Hahn 2005; Ravasi *et al*. 2012; Danza *et al*. 2018). Moreover, Lake Cadagno is considered an open living archive system to study microorganisms that have been key for the development of early life on Earth (Crowe *et al*. 2008; Canfield, Farquhar and Zerkle 2010; Dahl *et al*. 2010; Wirth *et al*. 2013).

The anoxygenic phototrophic sulfur bacteria community of Lake Cadagno plays an important ecological role with an high rate of ^14^CO_2_ fixation, as demonstrated by Camacho *et al*. (2001) who observed that, although representing only about 10% of the volume of the lake, up to 40% of the total ^14^CO_2_ photo-assimilation occurs in the chemocline. Further studies, using nanoscale secondary ion mass spectrometry (NanoSIMS; Musat *et al*. 2008; Zimmermann *et al*. 2015) and again ^14^C isotope quantitative assimilation (Storelli *et al*. 2013; Posth *et al*. 2017; Luedin *et al*. 2019), highlighted the different efficacy of individual bacterial populations. Indeed, among PSB, large-celled *Chromatium okenii* resulted the most effective photo-assimilator, while the dark chemo-assimilation (∼30%) was mostly carried out by small-celled PSB *Thiodictyon syntrophicum* and *Lamprocystis purpurea*. In all studies both GSB populations, although the most abundant numerically, resulted in a low assimilation of inorganic carbon. Recently, PSB *C. okenii* (Berg *et al*. 2019) and PSB *T. syntrophicum* (Luedin *et al*. 2019) have been shown to mediate micro-aerobic metabolism in the dark. High levels of dark inorganic carbon assimilation have also been observed in other stratified lakes (Hadas, Pinkas and Erez 2001; Casamayor 2010; Casamayor *et al*. 2012; Morana *et al*. 2016; Pjevac *et al*. 2019), suggesting that alternative metabolic pathways, such as chemosynthesis, are involved in the primary production of these environments.

Ecologically similar but phylogenetically distant, PSB and GSB represent a good example of convergent evolution, having evolved in the same natural environment exhibiting different evolutionary strategies (Stayton 2015). A major difference is the set of pigments necessary to harvest light, GSB possess chlorosomes structures conferring an higher affinity to low light intensity compared to PSB (Bryant and Frigaard 2006). A second important difference lies in the accumulation of sulfur globules (S^0^) inside and outside the cell for PSB and GSB, respectively (Frigaard and Dahl 2008). Sulfur globules can further be oxidized to sulfate when hydrogen sulfide becomes scarce in the environment. Recently, Danza *et al*. (2017) investigated the response to hydrogen sulfide oxidation of the anoxygenic photosynthetic sulfur bacteria in the chemocline of Lake Cadagno confirming the link between light-dependent H_2_S uptake rates and important changes in the intracellular complexity. Consequently, sulfide oxidation to elemental sulfur results to be light-intensity dependent (Hanson *et al*. 2013). Normally, microbial communities in sediments and anoxic zones organize according to the redox tower, this perspective suggests that chemical potential can be used to predict the activity of microbial communities and their biogeochemical impact in redox models. These are models that take into account the most important reduction–oxidation (redox) reactions involved in the metabolisms of microbial populations within ecosystem models, providing additional information on bacterial assimilatory and respiratory fluxes (Zakem, Polz and Follows 2020). Moreover, different evolutionary strategies can be found even within the same group. For example, large-celled PSB *C. okenii* possesses flagella, unlike small-celled PSB which rely on gas vacuoles for movement, conferring a rapid reaction time with a resulting better capacity to adapt to changes of environmental conditions. Once a minimum number of cells is reached, *C. okenii*’s phototactic but aerophobic movement (Pfennig 1962) produces a local remixing of water, triggering a process called bioconvection (Sommer *et al*. 2017; Sepúlveda Steiner, Bouffard and Wüest 2019). This phenomenon is strongly linked to the number of *C. okenii* cells and to the presence of oxygen, which, by stopping the cells, increases the density of the water that precipitates downwards. The presence of a similar number of *Cryptomonas* spp, a highly motile algae, in the upper part of the chemocline of Lake Cadagno, is also to be taken into account, although apparently it does not produce the same process of bioconvection (Rodrigo *et al*. 1999; Camacho *et al*. 2001; Camacho, Vicente and Miracle 2001). However, the ecological significance of this phenomenon for the population of *C. okenii*, and for the phototrophic sulfur bacteria community in general, remains still unknown.

The aim of this study is to investigate the primary production by comparing photo- and chemo-synthetic activity of three phenotypically different anoxygenic phototrophic sulfur bacteria species living in the chemocline of meromictic Lake Cadagno. We selected three strains such as the large-celled, motile by flagella PSB *C. okenii* strain LaCa, the small-celled PSB *T. syntrophicum* strain Cad16^T^ and the GSB *Chlorobium phaeobacteroides* strain 1VII D7, all previously isolated from Lake Cadagno and grown in pure cultures in the laboratory. First, we tested the CO_2_ photo- and chemo-assimilation *in situ* using dialysis bags and radioactive ^14^CO_2_ and compared the single population activity with the microbial community present in the chemocline. Then, in the laboratory, we investigated the capacity to photo-oxidize hydrogen sulfide at different light intensities and substrate concentrations, and we evaluated the role of low concentrations of oxygen in chemotrophic carbon fixation.

## MATERIALS AND METHODS

### Study site and sampling

Lake Cadagno is a crenogenic meromictic lake located in the Piora Valley at 1921 m above sea level, in the southern Swiss Alps (46°33′N, 8°43′E and depth approximately 21 m). In addition to surface water tributaries, there are sublacustrine springs that feed the lake with high-density water flowing through gypsum-rich (CaSO_4_) dolomite rock (CaMg(CO_3_)_2_). The high salinity and low temperature produce a high density, anaerobic bottom layer of water (monimolimnion), separated from the upper, limpid and aerobic water layer (mixolimiom). Between these two layers of water, lies a chemocline with steep gradients of chemical compounds such as hydrogen sulfide (HS^−^) and oxygen (O_2_).

Physicochemical parameters of the water column were determined using a multiparameter probe (CTD115M, Sea & Sun Technology, Trappenkamp, Germany) equipped with pressure (bar), temperature (°C), conductivity (mS/cm), dissolved oxygen (mg/L) and turbidity (FTU, Formazine Turbidity Unit) sensors. Moreover, the CTD is further equipped with a photosynthetically active radiation (PAR) sensor (LI-COR Biosciences, Lincoln, NE), detecting the spectral range (wave band) of solar radiation from 400 to 700 nm used by photosynthetic organisms in the process of photosynthesis, and a Blue-Green Algae Phycocyanin (BGAPC) sensor (Turner Designs, San José, CA).

For sampling at high vertical resolution (16 bits/s), water was pumped to the surface through a Tygon-tube (20 m long, inner diameter 6.5 mm and volume 0.66 L) at a flow rate of approx. 1.0 L/min using a diaphragm liquid pump (KNF Neuberger Inc., Trenton, NJ). Samples were stored in 50 mL falcon tubes in the dark and analyzed for microbiological (FISH and flow cytometry) and chemical (S^2−^, SO_4_^2−^ and total alkalinity) parameters within the following hour. For the sulfide analysis, 12 mL subsamples were immediately transferred to screw-capped tubes containing 0.8 mL of 4.0% ZnCl_2_ solution. For the sulfate and total alkalinity measures, 5 mL and 1 mL subsamples were processed, respectively. These solutions were analyzed colorimetrically using photometric Spectroquant^®^ kits (Merck, Schaffhausen, Switzerland).

### Phototrophic sulfur bacteria growth conditions

Phototrophic sulfur bacteria were grown in Pfennig's medium I (Eichler and Pfennig 1988), containing per liter: 0.25 g of KH_2_PO_4_, 0.34 g of NH_4_Cl, 0.5 g of MgSO_4_ × 7H_2_O, 0.25 g of CaCl_2_ × 2H_2_O, 0.34 g of KCl, 1.5 g of NaHCO_3_, 0.02 mg of vitamin B_12_ and 1.0 mL of trace elements solution SL10. The medium was prepared in a 2.0 L bottle using a flushing gas composition of 80% N_2_ and 20% CO_2_ according to (Widdel and Bak 1992) and was reduced by the addition of a Na_2_S × 9H_2_O solution a to a concentration of 1.0 mM S^2−^ and adjusted to a pH of approximately 7.1. All cultures were incubated at room temperature (22–23°C) and subjected to a light/dark photoperiod of 16/8 h with a light intensity of 136.4 μmol/m^2^/s PAR measured with a portable LI-180 Spectrometer (LI-COR Biosciences, Lincoln, NE) with incandescent 100 W bulbs emitting the entire visible spectrum (Figure S1, Supporting Information).

### Flow cytometry

Phototrophic sulfur bacteria in pure cultures were enumerated by flow cytometry (FCM) measuring chlorophyll-like autofluorescence particle events (Tonolla *et al*. 2017; Danza *et al*. 2018). A BD Accuri C6 cytometer (Becton Dickinson, San José, CA) equipped with two lasers (488 and 680 nm), two scatter detectors and four fluorescence detectors (laser 488 nm: FL1 = 533/30, FL2 = 585/40, FL3 = 670; laser 640 nm: FL4 = 675/25) was used for samples analysis. A total of two principal parameters were used for event characterization: forward scatter (FSC), which allows for the discrimination of cells by size, and 90° sideward scatter (SSC), which is proportional to the internal complexity of the cells. A threshold of 10 000 on FSC-H was applied to exclude most of the unwanted abiotic particles. Furthermore, to discriminate cells emitting autofluorescence due to chlorophyll and bacteriochlorophyll, a FL3-A >1100 threshold was applied on FL3 (red fluorescence). A volume of 50 μL was analyzed in triplicate for each sample at a 66 μL/min flow rate, and dilutions in Milli-Q water were performed for those samples with more than 10 000 events per second.

Green sulfur (GSB) and purple sulfur bacteria (PSB) colonizing the chemocline of Lake Cadagno were distinguished based on morphological characteristics (Danza *et al*. 2017). Large-celled PSB *C. okenii* LaCa, small-celled PSB *T. syntrophicum* Cad16^T^ and very small GSB *C. phaeobacteroides* 1VII D7 Lake Cadagno were well distinguished in a SSC vs FSC dot plot and gating on the respective populations permitted their quantification.

### Definition of the cellular biovolume

Cell volumes were calculated as in Tonolla *et al*. (2003). Biovolumes of bacterial cells were analyzed on images captured with a Zeiss Axiocam 305 color camera connected to a Zeiss Axio Scope A1 epifluorescence microscope (Zeiss, Oberkochen, Germany) using the ZEN 2.6 (blue edition) imaging software (Zeiss, Oberkochen, Germany). For each species, between 30 and 40 cells in the exponential phase were taken into account to determine the mean size.

### Measure of inorganic carbon assimilation using ^14^C

The ^14^C-radioisotope uptake experiment was carried out as previously described (Storelli *et al*. 2013).

Pure bacterial cultures enriched up to 10^6^ cells/mL (late exponential phase) were sealed in 50-cm-long dialysis bags (inflated diameter of 62.8 mm; Karl Roth GmbH Co. KG, Karlsruhe, Germany), that allowed for the free diffusion of molecules smaller than 20 kDa while preventing contamination of *in situ* incubated cultures by environmental bacteria. The final number of dialysis bags attached and incubated in the chemocline of Lake Cadagno was a sum of triplicate for every microorganism tested multiplied by two for both testing conditions of light and dark, for a total of 18. Moreover, the dialysis bags supporting frame was equipped with HOBO UA-002–64 Pendant passive data loggers (Onset Computer Corporation, MA) measuring relative light (Lux; 180–1200 nm) and temperature at 60 min intervals. A total of two sensors were placed on the top (11.6 m depth), one in the middle (11.8 m depth) and other two were positioned on the lower part (12.3 m depth) of the structure (Figure S2, Supporting Information). The HOBO logger values were analyzed after their retrieval at the end of the experiment.

**Figure 1.**
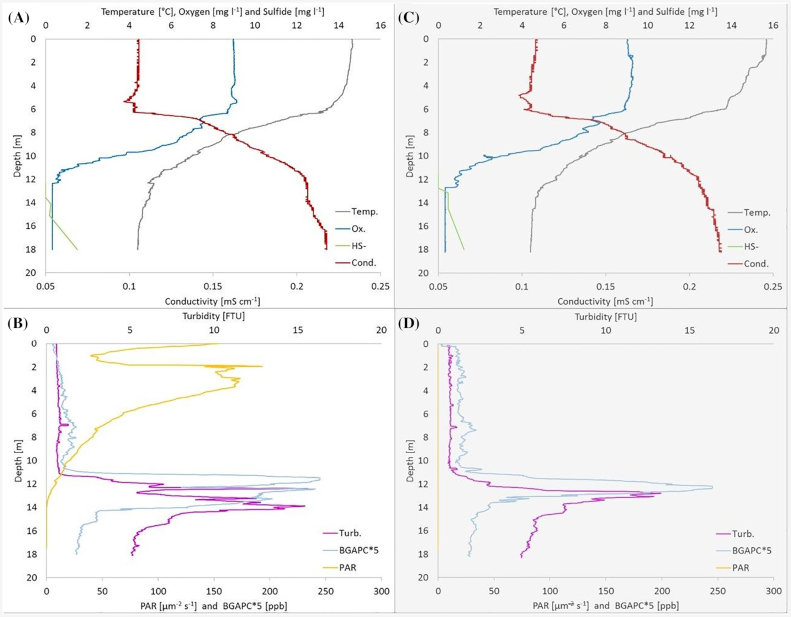
Vertical distribution of physicochemical profiles of the 22nd of August 2019. Physicochemical profiles of meromictic Lake Cadagno measured at 10:00 h **(A and B)** and at 22:00 h **(C and D)**. Measurements were taken from a platform (5 m long and 3 m wide) installed in the deepest part of the lake, after a CTD equilibration period of 5 min at 0.5 m depth. Top graphs (a) and (c) show profiles of temperature (grey), conductivity (red) sulfide (green) and oxygen concentration (blue), and bottom graphs (b) and (d) show profiles of turbidity (purple), phycocyanin (cyan) and light (yellow). Moving average was calculated on a factor of 10 elements for Turbidity and BGAPC data set of graphs b and d. Profiles of physicals parameters, such as temperature, conductivity, oxygen, hydrogen sulfide and light, are similar to past observation. However, biological parameters such as turbidity and BGAPC can change if measured at different period of the year.

The analysis of the inorganic carbon assimilation in the chemocline of Lake Cadagno was carried out on the 22nd of August 2019, after an acclimatization period of 6 weeks at between 11.78 and 12.28 m depth (from the 11th of July). The dialysis bags were retrieved, homogenized to avoid sample heterogeneities and dropped in 30 mL sealed bottles with 75 µL of radioactive ^14^C (NaH^14^CO_3_; 1.0 mCi; 8.40 mCi mM/L, 20 μCim/L; Cat. No. NEC-086S Perkin-Elmer, Zurich, Switzerland). On the 22nd of August 2019, at 10:00 h, triplicate of sealed 30 mL bottles filled with cultures coming from dialysis bags (cell counts are reported in Table 1) were incubated for 4 h at 11.8 m depth before acidification and bubbling (Gächter *et al*. 1979) to ensure a total loss of unbound radioactive carbon. The sealed bottles were filled to the top to ensure no air was left inside and exclude any possible effect of oxygen. The same procedure was repeated in dark conditions at 22:00 h. The total inorganic carbon assimilation was also measured in the upper part of the bacterial layer as a ‘positive control’ of cells activity, by collecting water from the top of the turbidity zone at 12.29 m (Fig. 1B, purple lines) and 12.65 m depth (Fig. 1D, purple line). The same water was also filtered with 0.22 μm filters to remove any microorganism in order to define a zero value, a ‘negative control’ of the ^14^C chemical background without biological activity.

**Table 1.** FISH and FCM (± standard deviation) quantification of PSB *C. okenii*, PSB *T. syntrophicum* and GSB *C. phaeobacteroides* in the chemocline of Lake Cadagno and in dialysis bags (22 August 2019).

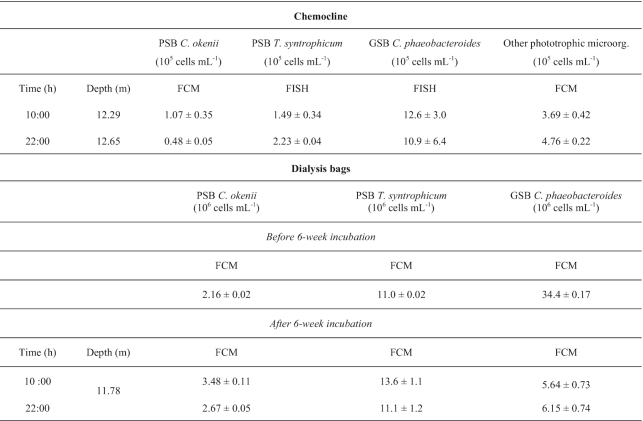

The same experiment was carried out in the laboratory under three different conditions, room temperature (RT; Pfennig medium at 22°C), 4°C (Pfennig medium at 4°C) and 5.0% oxygen (Pfennig medium at RT prepared with a flushing gas composition of 5% O_2_, 10% CO_2_ and 85% N_2_), using pure cultures with up to 10^6^ cells/mL. Each condition was incubated in the presence of light (approximately 1 h after 12 h of dark) and in the dark (approximately 1 h after 12 h of light). Triplicate of 125 mL sealed serum bottles with 50 mL of pure enriched culture were supplied with 100 µL of radioactive ^14^C and incubated 4 h before acidification and bubbling (Gächter *et al*. 1979).

Samples were divided in two, in one vial 7 mL of sample was acidified and bubbled for 1 h, while in another vial 7 mL was fixed with the addition of 10 mL of Ready Gel scintillation liquid (Ready GelTM; Beckman Coulter, Fullerton, CA). At the end of the bubbling period, 10 mL of Ready Gel scintillation were also added to the sample. The radioactivity of all samples was measured in a Beckman LS 6000 Scintillation Counter (Beckman, WS-BECKLS6).

The dissolved inorganic carbon concentration, needed for the calculation (Gächter *et al*. 1979), was determined with the CaCO_3_ Merck Spectroquant kit (No. 1.01758.0001) and the Merck spectroquant Pharo 100 photospectrometer (Merck & Cie, Schaffhausen, Switzerland).

### Light-dependent sulfide oxidation

PSB *C. okenii* LaCa, *T. syntrophicum* Cad16^T^ and GSB *C. phaeobacteroides* 1VII D7 isolated from Lake Cadagno chemocline were incubated in a single microrespiratory chamber system equipped with a sulfide gas sensor (SULF; range 0–300 μM, detection limit 0.3 μM; Unisense A/S, Aarhus, Denmark). The system was kept under continuous magnetic stirring at 200 rpm and at a constant temperature of 24°C. A total of four different H_2_S concentrations of 5, 10, 100 and 300 μM were tested by a sulfide pulse added before starting the measurement to achieve the desired initial concentration. The sensor detects the partial pressure of H_2_S gas, which is only one component of the total sulfide equilibrium system. Therefore, according to the following equation (Jeroschewski, Steuckart and Kuhl 1996):

\begin{eqnarray*} [{S^{2 - }}] = [{H_2}S]\left( {1 + \frac{{{K_1}}}{{[{H_3}{O^ + }]}}} \right) \end{eqnarray*}

where K_1_ is a reaction rate constant and [H_3_O^+^] is a proxy for the pH of the sample solution, the H_2_S concentrations correspond to S^2−^ concentrations of 0.02, 0.04, 0.40 and 1.20 mM.

A light/dark photoperiod (5 min/5 min) was applied using 100 W incandescent bulbs emitting the entire spectrum for a total of 90 min. The sample in the microrespiratory chamber was incubated under ten following increasing light intensities (0, 1, 2, 3, 5, 10, 20, 30, 40, 50 μmol/m^2^/s PAR). Light intensity was measured using a LI-193SA spherical quantum sensor (LI-COR Biosciences, Lincoln, NE) next to the incubation chamber.

Replicates incubated in the dark and a batch of medium incubated in the presence of light were used as negative controls with a concentration of 0.4 mM S^2−^.

The rate of sulfide metabolism for phototrophic sulfur bacteria is assumed to be dependent on both light and sulfide concentration according to the following equation:

\begin{eqnarray*} rate = k{[{{\rm{H}}_2}{\rm{S}}]^{\rm{a}}}[{\rm{light}}] \end{eqnarray*}

Assuming a constant initial sulfide concentration, the rate equation then becomes:

\begin{eqnarray*} {\rm{rate}} = k[{\rm{light}}] \end{eqnarray*}

The sulfide oxidation rate related to the respective light intensity was calculated through the integration of values at the start and the end of the light phase period. All different concentrations of sulfide were measured in triplicates.

The data were fitted to a Hill equation (Hill 1910) representing sulfide oxidation as a function of light intensity:

\begin{eqnarray*} V = \frac{{{V_{\max }}{{[light]}^n}}}{{{{(K)}^n} + [light]}} \end{eqnarray*}

Where *V*_max_ is the maximum rate of sulfide oxidation, *n* is the Hill coefficient which typically indicates substrate binding cooperativity in enzyme kinetics, or more generally a non-linear dose-response, *K* is a light half-saturation constant and [light] is the light radiation intensity. The equation was used to perform non-linear curve fitting to the experimental data using Levenberg–Marquardt algorithm.

In the fitting procedure, the baseline level of sulfide oxidation in the dark was added to the Hill equation and not varied throughout the fitting procedure.

### Nucleic-acid content determination

The relative total nucleic acid content for each sample was estimated through staining with SYBR Gold (Molecular Probes, Eugene, OR) with a final concentration of 1:10 000. The stained samples were incubated for 13 min at 37°C in the dark, and then 50 μL were measured using the flow cytometer (Becton Dickinson, San José, CA). Median fluorescence signal FL1-H (>1100) of gated populations allowed detecting changes in signal intensity due to the variation of nucleic acid content.

### Measure of intracellular ATP

The mean activity of microbial cells in pure cultures has been determined by quantifying the ATP present using the BacTiter-Glo Microbial Cell Viability Assay (Promega Corporation, Dübendorf, CH) and a BioOrbit 1253 luminometer (BioOrbit OY, Turku, Finland). The BacTiter-Glo reagent was prepared according to the manufacturer's instructions. The kit protocol recommends incubating an equal volume of reagent and sample (100 μL:100 μL) at room temperature for 5 min before recording the luminescence, expressed in Relative Light Units (RLU). The RLU values were converted to ATP concentrations using a calibration curve, measured with separate preparations of the ATP reagent.

### Fluorescent *in situ* hybridization (FISH)

The total cell number of PSB *T. syntrophicum* and GSB *C. phaeobacteroides* in the bacterial layer of the Lake Cadagno chemocline were identified and quantified using FISH with species-specific Cy3-labeled oligonucleotides, S453F (CCTCATGGGTATTARCCACAAGGCG) and CHLP 441 (AAATCGGGATATTCTTCCTCCAC), respectively. A total of 20 mL of lake water were filtered onto 0.2 μm polycarbonate filters, fixed for 30 min with a 4% paraformaldehyde solution and washed twice in PBS. Bacteria were then resuspended in 600 μL of PBS/EtOH 1:1. Both samples were observed in 2 and 5 µL aliquots of paraformaldehyde-fixed water samples (*n* = 3) spotted onto gelatin-coated slides (0.1% gelatin, 0.01% KCr(SO_4_)_2_; Glöckner *et al*. 1996). Hybridizations were performed as described by Zarda *et al*. (1997) with concomitant DAPI staining for total bacterial community quantification. The slides were treated with Citifluor AF1 (Citifluor Ltd., London, UK) and examined by epifluorescence microscopy using filter sets F31 (AHF Analysentechnik, Tübingen, Germany; D360/40, 400DCLP and D460/50 for DAPI) and F41 (AHF Analysentechnik; HQ535/50, Q565LP and HQ610/75 for Cy3). The microorganisms were counted at 1000× magnification in 40 fields of 0.01 mm^2^.

## RESULTS

### Physical and chemical analysis of Lake Cadagno water column

Measurements of the physicochemical parameters of Lake Cadagno water column were carried out on the 22nd August 2019. The profiles showed in Fig. 1 were taken at the beginning of both 4 h ^14^CO_2_ incubation periods in Lake Cadagno, at 10:00 h (Fig. 1A and B) and at 22:00 h (Fig. 1C and D).

The CTD analysis of the water column put in evidence the three distinct zones in the meromictic Lake Cadagno, the mixolimnion, well highlighted by profiles of oxygen in graphs (a) and (c) extending the aerobic water layer from the surface until approx. 12 m depth, where oxygen disappears (0.31 mg/L). In the mixolimnion profiles of oxygen, temperature and conductivity are homogenized down to approximately 6 m depth as a result of wind and convectively-driven surface mixing. Below the homogeneous zone, the temperature decreased from 14.6°C to its lowest value of 4.4°C, while conductivity increased from 0.108 to 0.205 mS/cm at 12 m of depth (Fig. 1, graph A and C). The values of temperature and conductivity did not change between day and night. The second zone is called chemocline (from approximately 12 to 14 m depth) and coincides with the beginning of the anaerobic layer, confirmed by the increase of the hydrogen sulfide concentration until a maximal value of 1.4 mg/L at 18 m of depth (Fig. 1A and C, green lines). Here the profile of conductivity remains constant in correspondence of the turbidity peak. In the chemocline the turbidity profile strongly increases to a maximal value up to 16.0 FTU (Fig. 1B and d, purple lines). During the day, the turbidity layer absorbed the light completely, and light did not penetrate below 14 m depth (Fig. 1B and D, yellow lines). The profile of light shows an uncommon behavior, with a steep reduction in the first 2 m, until approximately 4 m depth (Fig. 1B, yellow line). The measurement on the Lake Cadagno are normally carried out by the way of a platform (5 m long and 3 m wide) in the middle of the lake, where the depth is maximal (21 m depth). The CTD is lowered from the centre of the platform, to reduce oscillations as much as possible, producing this artificial profile derived from the shadow of the platform. A couple of meters before the complete disappearance of oxygen, the measure of BGAPC (Blue-Green Algae PhycoCyanin) reached the maximum value of approximately 50 ppb (Fig. 1B and D, cyan lines; values multiplied by 5). This peak of phycocyanin pigments highlights the presence of cyanobacterial-like microorganism close to the anaerobic water. The lower layer of the lake, called monimolimnion, from 14 m to the bottom, is dark, anoxic and with high-density water due to the low temperature and presence of dissolved salts.

### The anoxigenic phototrophic sulfur bacteria community

The turbidity peaks showed in Fig. 1 (B and D, purple lines) between 12 and 14 m depth were analyzed using flow cytometry (FCM) and fluorescent *in situ* hybridization (FISH) in order to identify our population of interest within the entire microbial community.

FCM analysis, using a red autofluorescence signal (laser 488, emission 670 nm), confirmed a high concentration of photosynthetic microorganisms in correspondence to the turbidity peaks (Fig. 1B and D). The top of the bacterial layer changed slightly during the day, at 10:00 h it was at 12.29 m and 22:00 h at 12.65 m of depth, while the number of photosynthetic cells at the top of the turbidity peak did not vary much with 6.34 × 10^5^ and 6.60 × 10^5^ cells/mL in the morning and in evening, respectively.

Big-celled *C. okenii* were directly identified and counted by FCM on SSC/FSC dot plots after positive FL3 fluorescence discrimination, revealing some difference in number between day and night, of 1.07 × 10^5^ and 4.88 × 10^4^ cells/mL, respectively. The remaining of the phototrophic community in the bacterial layer was composed mainly of PSB and GSB, as further confirmed by FISH analyses. GSB *C. phaeobacteroides* was the most abundant among the three strains, with day and night numbers of 1.26 × 10^6^ and 1.09 × 10^6^ cells/mL while PSB *T. syntrophicum* had a cell number of 1.49 × 10^5^ and 2.23 × 10^5^ cells/mL day and night, respectively (Table 1). All together this three species account for the 72.8% of the anoxygenic phototrophic sulfur bacteria community of Lake Cadagno, the remaining 27.2% being represented by other species not considered in this study, because phenotypically similar to small celled PSB *T. syntrophicum* (e.g. *Lamprocystis roseapersicina* and *purpurea;* and *Thiocystis chemoclinalis* and *cadagnonensis*) and to GSB *C. phaeobacteroides* (e.g. *C. chlatratiforme*) (Decristophoris *et al*. 2009).

### Day and night ^14^CO_2_ fixation in Lake Cadagno

After 4 h of incubation, the total fixed ^14^CO_2_ in the upper part of the bacterial layer (positive control) was 2672.9 ± 689.0 ^14^C nM/h in the presence of light and 264.3 ± 102.1 ^14^C nM/h in the dark. These values were transformed using the total number of phototrophic cells previously determined by FCM, with the result of a cell fixation rate of 210.7 ± 27.2 and 17.8 ± 4.1 ^14^C amol (attomoles)/cell/h, during day and night, respectively.

**Figure 2.**
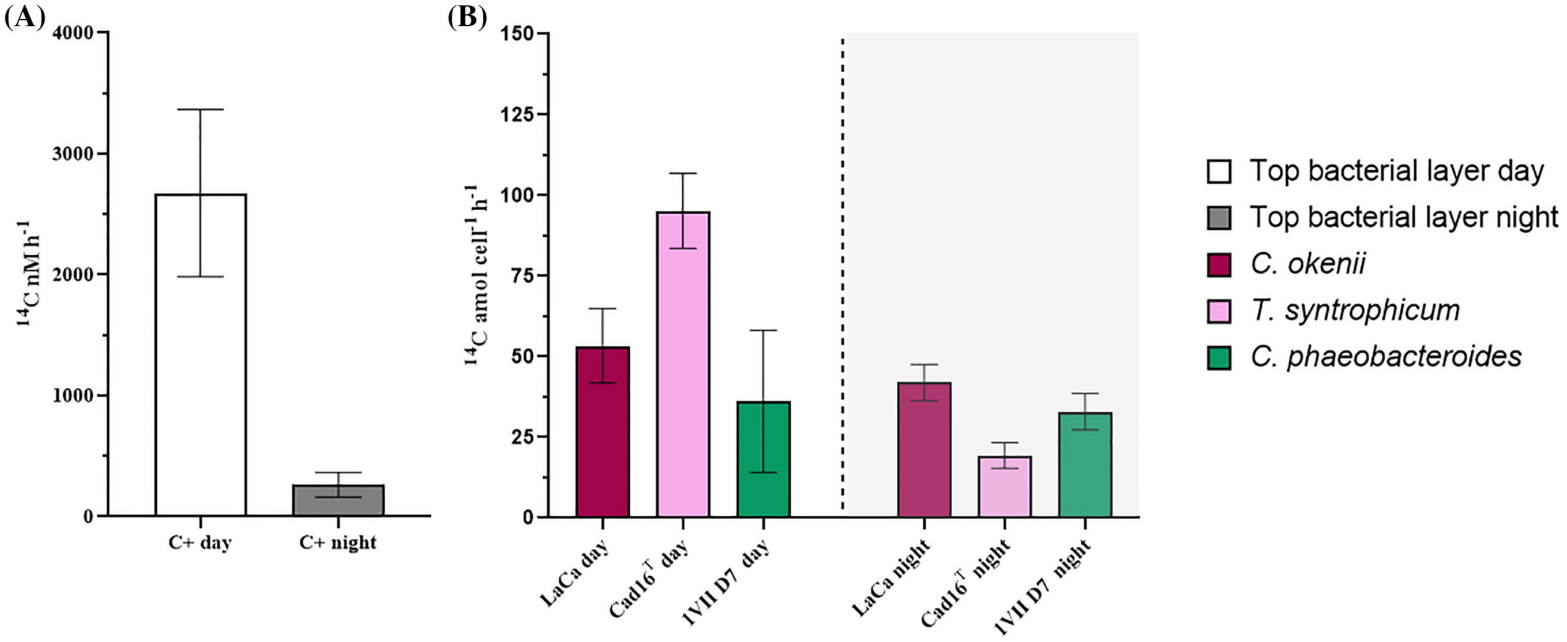
Day and night ^14^CO_2_ fixation in the Lake Cadagno using dialysis bags. **(A)** Total ^14^CO_2_ assimilated [nM/h] by the bacterial layer water sampled at 12.29 m during the day (white bar) and 12.65 m in the night (grey bar) from the chemocline of Lake Cadagno. The standard error of four replicates is showed on top of every bar. **(B)**  ^14^C-assimilation rates for all three microorganisms incubated in the dialysis bags, *C. okenii* LaCa, *T. syntrophicum* Cad16^T^ and *C. phaeobacteroides* 1VII D7. Error bars represent standard errors.

The uptake of inorganic carbon was also measured using pure cultures of the three different populations, the PSB *C. okenii* strain LaCa and *T. syntrophicum* strain Cad16^T^, and the GSB *C. phaeobacteroides* strain 1VII D7, incubated directly in the chemocline of Lake Cadagno in 50-cm-long dialysis bags (11.78–12.28 m depth). In the presence of light during the day (between 10:00 and 14:00 h) the most active ^14^CO_2_ assimilator microorganism was the small-celled *T. syntrophicum* Cad16^T^ with 95.2 ± 11.6 ^14^C amol/cell/h (Fig. 2B purple bar, white box). Big-celled *C. okenii* LaCa, assimilated around one half of *T. syntrophicum* with 53.4 ± 11.6 ^14^C amol/cell/h, while the least effective was the GSB *C. phaeobacteriodes* 1VII D7 with 36.1 ± 21.9 ^14^C amol/cell/h (Fig. 2B violet and green bars, white box). During the night without any light irradiation (between 22:00 and 02:00 h), the situation of ^14^CO_2_ fixation for *C. okenii* LaCa and *C. phaeobacteroides* 1VII D7 was similar to that observed during the day in presence of light, with 42.0 ± 5.6 and 32.9 ± 5.6 ^14^C amol/cell/h (Fig. 2B violet and green bars, grey box). Instead, the assimilation rate of *T. syntrophicum* Cad16^T^ in the night was five times lower, 19.3 ± 4.0 ^14^C amol/cell/h, compared to the day (Fig. 2B purple bar, grey box).

From the beginning of the pre-incubation period (from the 11th of July to 22nd of August), then during (the 22nd and 23rd of August) and after the the experiment days (until the 27th of September) the temperature and light were recorded by HOBO loggers attached on the structure holding the dialysis bags (top, middle and bottom). Temperature increased over the summer, varying from 4.5 up to 6°C at 11.8 m of depth.. The high peaks of temperature correspond to the time the dialysis bags were collected, for the ^14^C experiment (Figure S3, Supporting Information). Light intensity ranged from 0 during the night to higher values during the day of around 7.6, 3.8 and 2.4 μmol/m^2^/s PAR on the top, in the middle and at the bottom of the incubation structure, respectively.

This experiment highlighted the importance of photosynthesis in the CO_2_ fixation process for the bacterial chemocline community of Lake Cadagno with a yield 10 times higher than at night. As for pure cultures, only PSB *T. synthrophicum* showed similar behavior, while *C. okenii* and *C. phaeobacteroides* did not seem to show a big difference between day and night.

### Role of oxygen in the ^14^CO_2_ fixation

**Figure 3.**
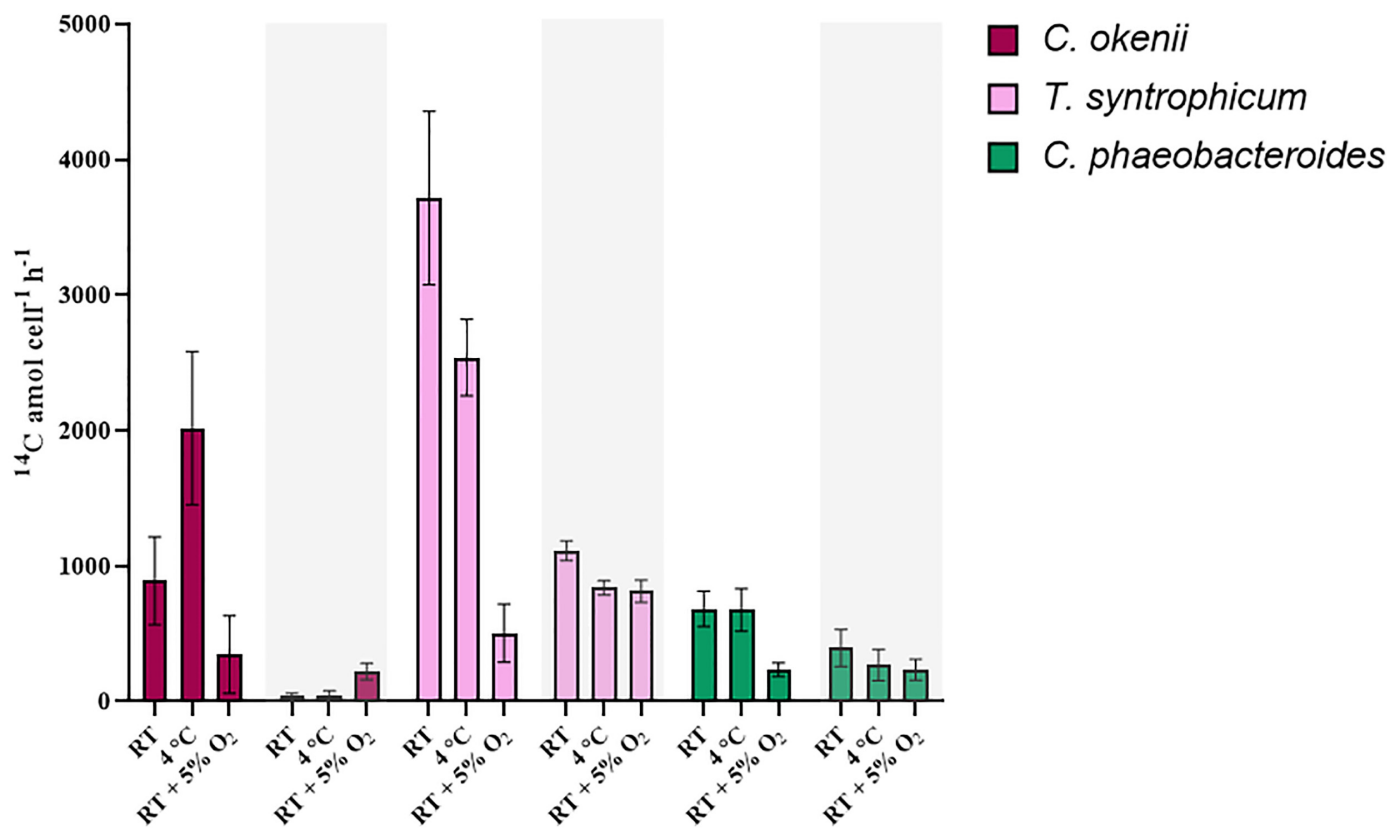
^14^CO_2_ assimilation under laboratory conditions. Inorganic carbon fixation [^14^C amol/cell/h] by pure cultures of PSB *C. okenii* LaCa, PSB *T. syntrophicum* Cad16^T^ and GSB *C. phaeobacteroides* 1VII D7 isolated from Lake Cadagno in the presence of light and in the dark (grey areas). RT (room temperature) was 22°C. Fixation of ^14^CO_2_ was quantified during 4 h in liquid autotrophic cultures. Standard errors are shown as error bars.

Inorganic carbon fixation was further analyzed in the laboratory evaluating two environmental conditions such as low temperature (around 4°C) and the presence of microaerophily (around 5% of O_2_). The oxygen was added in order to verify the possibility of PSB chemotrophic microrespiration suggested in recent studies (Berg *et al*. 2019; Luedin *et al*. 2019). The low temperature measurement, similar to the natural environment, has been selected to verify the possibility of artifacts related to the higher temperatures used in growing PSB and GSB in the laboratory. In the 5% O_2_ incubation, no sulfide was present in the culture bottles whereas in the other incubation conditions (RT and 4°C) sulfide concetration was similar to that observed in the chemocline of the lake. In Fig. 3, both environmental conditions are compared to the standard growth conditions (see materials and methods) in presence of light and in the dark. In general, for all situations tested, the fixation of ^14^CO_2_ was higher compared to the values showed in the previous experiment where fixation was analyzed in the chemocline of Lake Cadagno (Fig. 2), except for the two dark fixation rates without oxygen of *C. okenii* LaCa.

*C. okenii* LaCa cultures showed almost no fixation in the dark, except for low activity in the presence of 5% O_2_, where the amount of CO_2_ assimilated settled at 220.6 ± 61.2 ^14^C amol/cell/h. Interesting, the activity almost doubled for samples incubated at low temperatures (4°C) compared to room temperature, 2017.6 ± 565.2 and 890.4 ± 323.7 ^14^C amol/cell/h, respectively. Similarly to the dialysis bags experiment (Fig. 2), *T. syntrophicum* Cad16^T^ showed the highest values of CO_2_ fixation also in laboratory conditions, both in the presence of light and in the dark. Its rates of 3715.9 ± 640.2 and 2538.4 ± 283.1 ^14^C amol/cell/h under light exposure at room and low-temperature conditions, respectively, were the most significant amount of CO_2_ fixed recorded in the experiment. As for *C. okenii*, in the presence of 5% oxygen, photo-assimilation strongly decreased to a value of 504.5 ± 215.2 ^14^C amol/cell/h. *T. syntrophicum* strain Cad16^T^ is known to be able to fix CO_2_ also in the absence of light (Peduzzi *et al*. 2012; Storelli *et al*. 2013, 2014), explaining its strong dark activity. GSB *C. phaeobacteroides* 1VII D7 showed a similar activity around 700 ^14^C amol/cell/h in the presence of light but without oxygen, where activity is strongly reduced. In the dark, the fixation rate was low, around 200 ^14^C amol/cell/h, also in presence of oxygen, confirming GSB's strictly anaerobic behavior (Frigaard and Dahl 2008; Haas *et al*. 2018).

Therefore, as already observed in the field experiment, photosynthesis was more efficient in terms of ^14^CO_2_ fixation in the laboratory experiment as well, with both PSB being more performing than *C. phaeobacteroides*. The presence of oxygen strongly reduced photosynthesis, but had no effect for chemosynthesis, except for an increase in activity for *C. okenii*.

### Photo-oxidation of sulfide at different light intensities

Following the ^14^CO_2_ assimilation assessment, the light-dependent H_2_S oxidation rates of both dialysis bags and laboratory cultures was also tested. Growth rates of the laboratory grown three phototrophic sulfur bacteria species analyzed have been determined by FCM (Figure S4, Supporting Information). Bacterial cells were grown to their late exponential phase, at a concentration of approx. 4.0 × 10^6^ cells/mL for *C. okenii* LaCa and approx. 1.0 × 10^7^ cells/mL for both *T. syntrophicum* Cad16^T^ and *C. phaeobacteroides* 1VII D7, then the effect of light intensity and different hydrogen sulfide concentration was tested. The oxidation rate of each strain and condition has been compared taking into account the maximal velocity of the oxidation process (*V_max_*) and light half-saturation constant (*K*; Table 2).

**Table 2.** Comparison of kinetic parameters, *V_max_* (μmol/cell/h) and K (μE/m/s), derived from enzyme activity model.

S^2−^ (mM)	*C. okenii* LaCa		*T. syntrophicum* Cad16^T^		*C. phaeobacteroides* 1VII D7	
	*V_max_*	*K*	*V_max_*	*K*	*V_max_*	*K*
Dialysis bags cultures						
0.02	2.29 × 10^−8^	4.41	1.57 × 10^−8^	235.05	2.66 × 10^−10^	3.54
0.04	5.42 × 10^−9^	10.64	1.21 × 10^−8^	19.31	9.20 × 10^−10^	90.62
0.4	7.19 × 10^−9^	9.27	3.23 × 10^−9^	14.52	1.51 × 10^−8^	598.64
Laboratory cultures						
0.04	2.53 × 10^−9^	3.40	3.06 × 10^−9^	4.89	1.75 × 10^−9^	3.15
0.4	3.42 × 10^−8^	11.07	3.04 × 10^−9^	6.33	2.57 × 10^−9^	8.92
1.2	9.72 × 10^−8^	32.01	7.15 × 10^−9^	11.14	8.00 × 10^−9^	5.38

**Figure 4.**
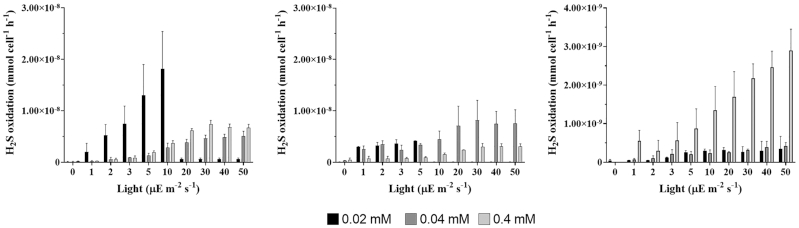
Light-dependent sulfide oxidation in dialysis bags cultures. Sulfide oxidation rate [μmol/cell/h] vs light [μE/m^2^/s] for dialysis bags cultures of **(A)**  *C. okenii* LaCa, **(B)**  *T. syntrophicum* Cad16^T^ and **(C)**  *C. phaeobacteroides* 1VII D7 at different light intensities. Error bars represent standard deviation (*N* = 3). If no error bars are shown, SD was smaller than the symbols used.

**Figure 5.**
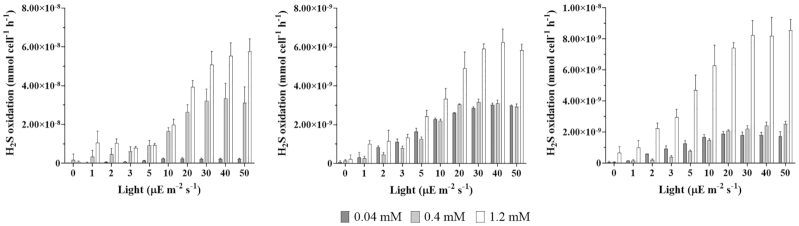
Light-dependent sulfide oxidation in laboratory cultures. Sulfide oxidation rate [μmol/cell/h] vs light [μE/m^2^/s] for laboratory cultures of **(A)**  *C. okenii* LaCa, **(B)**  *T. syntrophicum* Cad16^T^ and **(C)**  *C. phaeobacteroides* 1VII D7 at different light intensities. No oxidation was observed at the concentration of 0.02 mM S^2−^. Error bars represent standard deviation (*N* = 3). If no error bars are shown, SD was smaller than the symbols used.

Under both conditions, in all the three strains, the increase in light intensity irradiation (from 0 to 50 μE m^2^/s) resulted in an increase in the sulfide oxidation rates, confirming the positive effect of light irradiance on the photosynthetic process (Figs 4 and 5). A saturation effect on the S^−2^ consumption is also visible. For this reason, the photo-oxidation activity is measured for different increasing sulfide concentrations. The kinetic parameters considered for the strains are summarized in Table 2.

#### Dialysis bags cultures

Cultures of *C. okenii* LaCa reached their maximal sulfide oxidation rate of 2.29 × 10^−8^ μmol/cell/h with a starting S^2−^ concentration of 0.02 mM, with a corresponding light intensity constant K of 4.41 μE/m^2^/s (Fig. 4A and Table 2). The lowest sulfide oxidation rate of *C. okenii* was of 5.42 × 10^−9^ μmol/cell/h under the concentration of 0.04 mM S^2−^, with a corresponding K value of 10.64 μE/m^2^/s (Fig. 4A and Table 2). *T. syntrophicum* Cad16^T^ showed similar values with both 0.02 and 0.04 mM S^2−^, with an oxidation rate of 1.57 × 10^−8^ μmol/cell/h with K of 235.05 μE/m^2^/s and 1.21 × 10^−8^ μmol/cell/h with K of 19.31 μE/m^2^/s, respectively. Differently to *C. okenii*, *T. syntrophicum* had its minimal sulfide uptake of 3.23 × 10^−9^ μmol/cell/h under the 0.4 mM concentration, with a K of 14.52 μE/m^2^/s. *C. phaeobacteroides* 1VII D7 showed the lowest sulfide oxidation rates under every concentration, with a maximal oxidation rate of 1.51 × 10^−8^ μmol/cell/h and a K value of 598.64 μE/m^2^/s under the concentration of 0.4 mM S^2-^ (Fig. 4C and Table 2). In all cases, a saturation effect produced by the light intensity increase has been observed. The highest sulfide concentration of 1.2 mM was not analysed for dialysis bags cultures because too high compared to that encountered in the natural environment.

#### Laboratory cultures

In *C. okenii* LaCa cultures, the increase in light intensity irradiation resulted in marked changes in the sulfide oxidation rates, with little variation among analytical replicates. Maximal sulfide oxidation rates of 9.72 × 10^−8^ μmol/cell/h was reached with a starting S^2−^ concentration of 1.2 mM, with a corresponding light intensity constant K of 32.01 μE/m^2^/s (Fig. 5A and Table 2). The lowest sulfide oxidation rate of *C. okenii* was of 2.53 × 10^−9^ μmol/cell/h under the initial concentration of 0.04 mM S^2−^, with a corresponding K value of 3.40 μE/m^2^/s (Fig. 5A and Table 2). *T. syntrophicum* Cad16^T^ showed a similar behavior with both 0.04 and 0.4 mM S^2−^, with an oxidation rate of 3.06 × 10^−9^ μmol/cell/h with K of 4.89 μE/m^2^/s and 3.04 × 10^−9^ μmol/cell/h with K of 6.33 μE/m^2^/s, respectively. Similarly to *C. okenii*, *T. syntrophicum* reached its maximal sulfide uptake of 7.15 × 10^−9^ μmol/cell/h under the 1.2 mM concentration, with a K of 11.14 μE/m^2^/s. *C. phaeobacteroides* 1VII D7 showed sulfide oxidation rates comparable to *T. syntrophicum* under every concentration, with a maximal oxidation rate of 8.00 × 10^−9^ μmol/cell/h and a K value of 5.38 μE/m^2^/s under the concentration of 1.2 mM S^2−^ (Fig. 5C and Table 2). As observed with the dialysis bags cultures, the light intensity increase produced a saturation effect. In the control microcosms kept in the dark, as well as in control with no cells exposed to light, no sulfide oxidation occurred (Figure S5, Supporting Information). At the concentration of 0.02 mM S^2−^ no oxidation activity was observed in laboratory cultures.

Essentially, dialysis bag cultures seem to be more adapted, and therefore photosynthetically efficient, to low light intensities and sulfide concentrations.

#### Light-dependent intracellular variations

**Figure 6.**
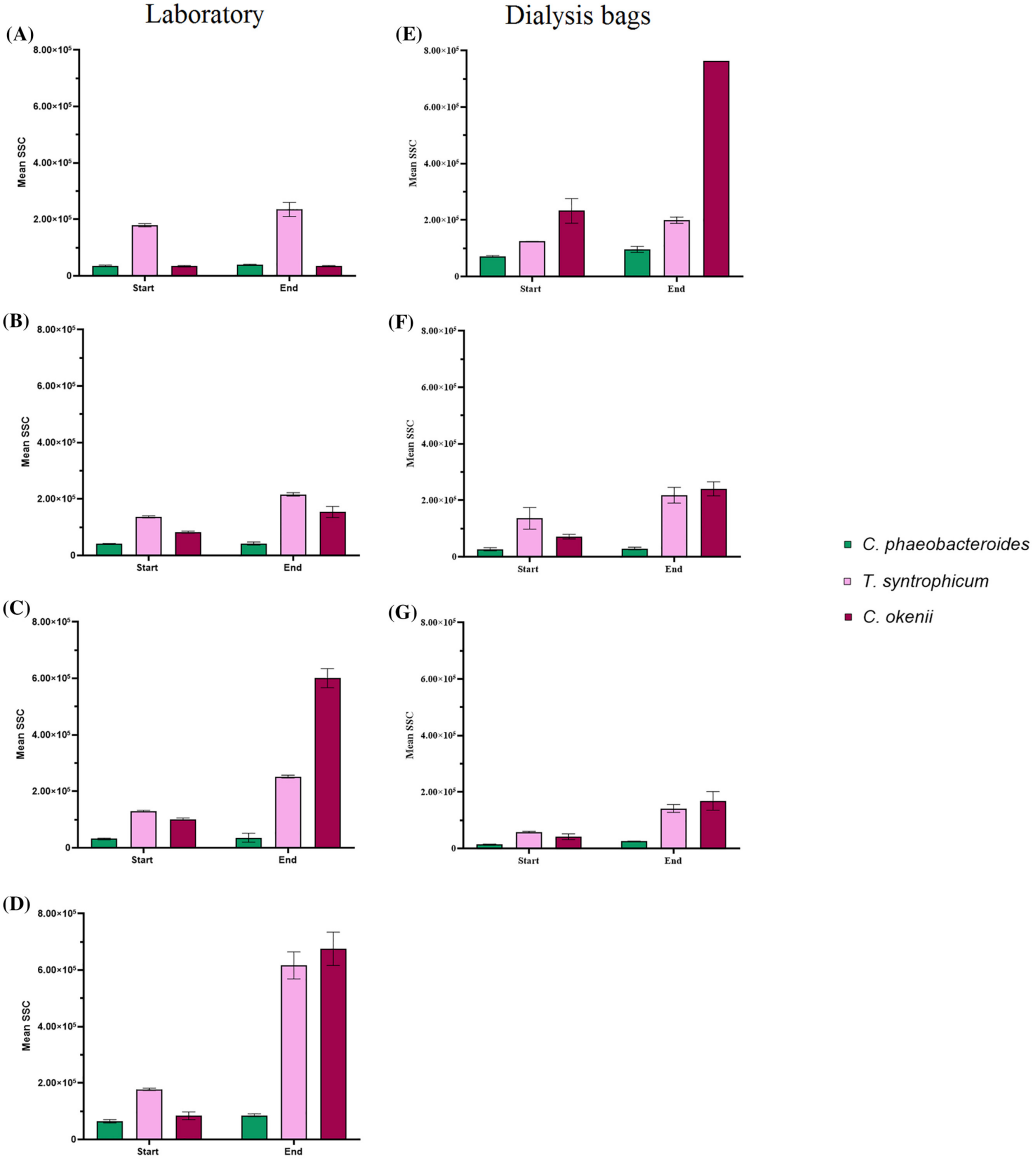
Flow cytometry cellular dynamics in anoxygenic photosynthetic sulfur bacteria. Mean SSC for laboratory and dialysis bags cultures before (Start) and after (End) the sulfide oxidation test under **(A and E)** 0.02 mM S^2^−^^ (no H_2_S oxidation observed in laboratory cultures), **(B and F)** 0.04 mM S^2−^, **(C and G)** 0.4 mM S^2−^, **(D)** 1.2 mM S^2^−^^. Error bars represent standard deviation (*N* = 3). If no error bars are shown, SD was smaller than the symbols used.

As a conquence to the exposure to different light intensities and sulfide concentrations, PSB *C. okenii* LaCa and *T. syntrophicum* Cad16^T^ showed significant dynamic cellular complexity variations during the photosynthetic activity. These changes correlated with variations in SSC signal intensity (Fig. 6), due to the formation of sulfur globules (Danza *et al*. 2017). For *C. okenii* laboratory cultures, with initial concentrations of 0.04 and 0.4 mM S^2−^, sulfide consumption correlated with an increase of SSC (Δ approx. 7.1 × 10^4^ and 5.0 × 10^5^, respectively) between the start and the end of the experiment. The 1.2 mM S^2−^ concentration corresponded to the maximum increase in SSC (Δ approx. 6.0 × 10^5^). In dialysis bags cultures of *C. okenii*, an increase in the concentration of sulfide (0.02–0.4 mM S^2−^) corresponded to a progressively lower Δ of approx. 5.3 × 10^5^, 1.7 × 10^5^ and 1.3 × 10^5^, respectively (Fig. 6E–G). On the contrary, the FCM forward scatter (FSC) signal did not vary significantly during photo-oxidation of sulfide, indicating no change in the cell size (Figure S6, Supporting Information). Small-celled PSB *T. syntrophicum* Cad16^T^ showed similar behavior in both laboratory and dialysis bags cultures, although with a less pronounced increase in the SSC signal over the measurements. The maximum increases in SSC signal (Δ approx. 4.4 × 10^5^ and 8.3 × 10^4^) were observed with an initial S^2−^ concentration of 1.2 mM and 0.4 mM, for laboratory and dialysis bags cultures, respectively (Fig. 6D and G).

*C. phaeobacteroides* 1VII D7 laboratory and dialysis bags cultures showed no effect on both SSC or FSC (Fig. 6 and Figure S6, Supporting Information).

Sulfur globules are not the only compounds supposed to alter the cellular complexity and, as a consequence, the SSC parameter. SYBR Gold staining to detect changes in nucleic-acid content of the three strains did not show any particular variation between the start and the end of the experiment (Figure S7, Supporting Information), indicating no changes in the concentration of double- or single-stranded DNA or RNA following the exposure to S^2^−^^ and light.

Together with nucleic acids, the formation of glycogen was estimated by way of the measure of the cellular ATP concentration. PSB *C. okenii* showed a slight increase in the ATP production after the exposure to light under all the S^2−^ concentrations tested (Figure S8a and d, Supporting Information). For PSB *T. syntrophicum* a substantial increase in ATP was observed under the 1.2 mM S^2−^, with small differences among the other S^2−^ initial concentrations and incubation conditions, although with a significantly lower ATP concentrations compared to the other strains (Figure S8b and e, Supporting Information). Similarly to PSB, we observed an increase in the ATP concentration for GSB *C. phaeobacteroides* in both laboratory and dialysis bags cultures for all the hydrogen sulfide concentrations tested (Figure S8c and f, Supporting Information).

As already shown in the past (Danza *et al*. 2017), the oxidation of sulfide correlates with the production of intracellular sulfur globules of PSB, resulting in an increase of the SSC parameter. At low concentrations of H_2_S only cultures from dialysis bags showed a substantial increase in SSC, further confirming their adaptation to lower concentrations of sulfide.

## DISCUSSION

### Physicochemical and biological properties of Lake Cadagno

In meromictic Lake Cadagno, CTD profiles observed on the 22nd of August 2019 were in line with past measurements of the same period (Storelli *et al*. 2013; Danza *et al*. 2017; Luedin *et al*. 2019). The profiles of sulfide concentration during day and night are almost similar (Fig. 1A and C; green graphs), however in presence of light the beginning of sulfide and the disappearance of oxygen (Fig. 1A and C, blue graphs) had a distance of about 2 m, in correspondence with the turbidity peak, which highlights phototrophic activity with consequent oxidation of HS^−^ (Fig. 1B and D; purple graphs). This turbidity peak coincides with the maximal density of anoxygenic phototrophic sulfur bacteria community, as confirmed by FCM and FISH analyses (Table 1). This bacterial layer changed in depth between the two observations at 10:00 h and 22:00 h lowering its depth of 36 cm, from 12.29 m to 12.65 m, respectively. This short-term difference is likely caused by gravity-based oscillations within the lake water column due to internal waves, also called internal seiches, typical of stratified water bodies where differences in temperature and/or salinity determine density variations with depth (Imboden and Wüest 1995; Bouffard and Boegman 2012; Bouffard and Wüest 2019).

Within the bacterial layer correlating with the turbidity peak, we observed an almost uniform trend in the temperature and salinity profiles below the oxic-anoxic interface (Fig. 1A and C; grey and red graphs). This uniformity has been considered an indication of mixing, in this case bioconvection, as it has been demonstrated to be caused by the movement of PSB *C. okenii* (Sommer *et al*. 2017). In fact, *C. okenii* is capable of swimming using a flagellum in search of the optimal conditions of light (phototaxis), and sulfide (positive gradient) and oxygen (negative gradient) concentration (Pfennig 1962; Egli *et al*. 2004; Sommer *et al*. 2017).

### CO_2_ assimilation in the chemocline of Lake Cadagno

In this study, measurement of *in situ*  ^14^C assimilation from dialysis bags further confirmed a strong total inorganic carbon fixation rate in the chemocline of Lake Cadagno, especially during the day, which showed a 10-fold higher values in the presence of light (Fig. 2A). The CO_2_ assimilation rate recorded in the dark was of the same order of magnitude as that found recently by Luedin *et al*. (2019), but much lower compared to earlier studies using dialysis bags (Storelli *et al*. 2013) and punctual *in situ* analysis (Camacho *et al*. 2001). In both studies, measurements were taken in September, when *C. okenii* population normally declines, making way for the development of small-celled PSB populations (Danza *et al*. 2018), known to be able to fix CO_2_ in the dark (Storelli *et al*. 2013). In our case the dominance of *C. okenii* was also confirmed by the presence of bioconvection, as shown by temperature and conductivity profiles (Fig. 1A and C; blue and green graphs), normally absent in September. Moreover, the dark fixation measurement was carried out during the day in parallel to the phototrophic fixation by the aid of opaque bottles, so ongoing phototrophic activity might have influenced the results increasing the dark fixation values.

In the presence of light, our values were in between higher values recorded by Storelli *et al*. (2013) and Camacho *et al*. (2001) and lower values observed by Musat *et al*. (2008) and Luedin *et al*. (2019). In 2019 we were confronted with a particular weather situation with a very cold Spring that caused ice-cover on Lake Cadagno to thaw only at the beginning of June (usually at the end of April). This late Winter condition and late Summer stratification negatively influenced the development of chemocline microorganisms highlighted during the season by much lower turbidity in the bacterial layer than normal (15–20 instead of 35–40 FTU). The lack of a complete development of the bacterial community can partially explain the total lower ^14^CO_2_ assimilation observed during our experiment. This may also be due to the seasonal variations observed in the phototrophic sulfur bacteria community composition (Danza *et al*. 2018). PSB *C. okenii* is present in high concentrations in mid-Summer (July), when bioconvection is more intense (Sommer *et al*. 2017), while other PSB and GSB are less abundant. The decrease in *C. okenii* population observed towards the end of Summer (late September), when bioconvection ceases, correlates with an increase in small-celled PSB and GSB (Danza *et al*. 2018).

Among the phototrophic sulfur bacteria strains selected for this study, PSB *T. syntrophicum* Cad16^T^ had the highest CO_2_ fixation activity in the presence of light, almost double compared to the value of PSB *C. okenii* LaCa and GSB *C. phaeobacteroides* 1VII D7 (Fig. 2B). This observation confirms data from previous studies (Storelli *et al*. 2013, 2014; Luedin *et al*. 2019) about the inorganic carbon fixation effectiveness of *T. syntrophicum* Cad16^T^ during daytime. The higher inorganic ^14^C-uptake rates we registered during the day compared to those observed at night suggests that PSB can actively carry out photosynthesis even at the low light intensities of the chemocline (between approx. 0.8 and 6.7 μmol/m^2^/s PAR), as described by Fischer *et al*. (1996).

Despite being the most abundant strain but the smallest in cell size, GSB *C. phaeobacteroides* 1VII D7, was the least efficient CO_2_-fixing strain in the light. Following this observation, CO_2_ assimilation rates were normalized to the average cell volume of 62 ± 14 μm^3^ calculated for *C. okenii* and 4.2 ± 1.3 μm^3^ and 0.8 ± 0.3 μm^3^ obtained for *T. syntrophicum* and *C. phaeobacteroides*, respectively. This resulted in *C. phaeobacteroides* becoming far more efficient than both PSB strains (data not shown). However, rates per cell volume do not reflect the true ecological relevance of each strain in the chemocline because surface to volume ratio is higher for small cells, therefore overestimating *C. phaeobacteroides* and *T. syntrophicum*’s contribution at the expense of large-celled *C. okenii*. For this reason, number of cells rather than volume was considered in this study.

The average *C. phaeobacteroides* carbon uptake rate of 36.1 ± 21.9 ^14^C amol/cell/h during daytime is comparable to that previously observed in Lake Cadagno for the other GSB *Chlorobium clathratiforme* strain Cad4DE living in Lake Cadagno (Musat *et al*. 2008; Storelli *et al*. 2013). This further suggests that GSB are generally less effective in assimilating CO_2_ than PSB in the presence of light. On the other hand, was surprising the fact that *T. syntrophicum* had the lowest CO_2_ assimilation rate in the absence of light (Storelli *et al*. 2013), but in this study the dark fixation phase was performed during the night (between 22:00 h and 02:00 h) and not during the day with opaque bottles. It is likely that the effectiveness of PSB in fixing CO_2_ differs during the all day (24 h) not only according to the presence or absence of light, as already suggested by a 24 h experiment under laboratory condition (Storelli *et al*. 2013), and for other microorganisms (Hancke *et al*. 2015; Sandrini *et al*. 2016; Alcamán-Arias *et al*. 2018). Therefore, the choice of a 4h-time span during the day for the ^14^CO_2_ fixation analyisis is critical for reproducibility, and comparison with other studies, of the presented results.

Dark activity of *C. okenii* in Lake Cadagno has previously been described by Berg *et al*. (2019), who observed a significant amount of C-fixation in the absence of light. It is known that some PSB species possess the capacity of microaerobic respiration (Camacho, Vicente and Miracle 2000; Casamayor, García-Cantizano and Pedrós-Alió 2008; Peduzzi *et al*. 2011; Luedin *et al*. 2019), and therefore it may well be possible that *C. okenii* showed chemoautotrophic growth on oxygen, in the absence of light. This is corroborated by our results from the laboratory experiment, where we observed an increased in *C. okenii*’s dark fixation in the presence of oxygen. This consideration is also supported by the fact that, on the day of the test (at 10:00 h), the chemocline resulted partly oxygenated with around 0.57 mg O_2_/L (36 µM) in presence of light and 0.95 mg O_2_/L (59 µM) in the dark at the depth where the dialysis bags were positioned. This small amount of oxygen probably diffused from the oxic upper layer produced during the day by photosynthetic microplankton, such as cryptomonads and other cyanobacteria-like microorganisms (Fig. 1B and D, cyan graphs), which are known to be present at the oxic-anoxic boundary (Camacho *et al*. 2001).

The CO_2_ fixation of *C. phaeobacteriodes* 1VII D7 is similar both in the day and at night, confirming earlier observations of members of the *Chlorobium* genus being able to assimilate inorganic carbon in the absence of light (Habicht *et al*. 2011). Since chemoautotrophic growth of GSB has not been reported so far, this dark activity might be due to the fermentation of organic compounds, such as glycogen, stored in the cell and not to the presence of oxygen (Casamayor, García-Cantizano and Pedrós-Alió 2008; Habicht *et al*. 2011; Casamayor *et al*. 2012). In fact, it has been proposed that glycogen can be consumed in the dark as an energy source via a mixed acid fermentation where CO_2_ is required to convert phosphoenol pyruvate to oxaloacetate, which is then transformed to succinate using the reverse tricarboxylic acid cycle (rTCA; Sirevåg 1995; Overmann 2006; Habicht *et al*. 2011).

### CO_2_ assimilation under controlled laboratory conditions

The ^14^CO_2_ assimilation rate of pure cultures was also analyzed under laboratory conditions, testing the low temperature and low concentration of oxygen typical of the chemocline of Lake Cadagno. Overall, the CO_2_ uptake rates were higher compared to the values obtained in the *in situ* experiment, where fixation was analyzed in the chemocline of the lake (Fig. 2). This was due to the nearly 40-fold higher light intensity used in the laboratory (136.4 μmol/m^2^/s PAR) compared to that of the chemocline (approximately 3.75 μmol/m^2^/s PAR on average), in fact *in situ* and *in vitro* maximum ^14^CO_2_ fixation rates differ by approximately the same factor.

*T. syntrophicum* Cad16^T^ showed the highest values of CO_2_ fixation also under the different laboratory conditions, both in the presence of light and in the dark (Fig. 3, pink bars). Chemo-assimilation of inorganic carbon in the presence of low oxygen concentration was similar to both other conditions, suggesting the ability to survive under microaerophilic conditions, although the chemosyntesis process is not only related to the presence of oxygen (Peduzzi *et al*. 2012; Luedin *et al*. 2019). Past proteomics analysis highlighted the presence of overexpressed enzymes part of the beta-oxidation of fatty acid and polyhydroxybutyrate (PHB) granules that should produce reducing power in the form of NAD(P)H, then potentially used in the CO_2_ fixing process (rTCA or/and dicarboxylate/4-hydroxybutyrate cycle or others) during the dark period (Storelli *et al*. 2014).

During the day, *C. okenii* was also a strong CO_2_ photo-assimilator, particularly at low temperatures, while in the dark the presence of oxygen strongly enhance the chemo-assimilation (Fig. 3, red bars in the grey box). Our observations, both *in vitro* and *in situ*, show that, in the presence of low levels of oxygen, dark CO_2_ fixation activity of *C. okenii* LaCa is present, thereby confirming that these bacteria can take energy in the dark from aerobic respiration but with much lower efficiency, in accordance with recent results (Berg *et al*. 2019). The fact that *C. okenii* and other members of the family *Chromatiaceae* are capable of aerobic respiration (Kampf and Pfennig 1980) is also suggested by the presence of several oxygen-dependent enzymes such as cytochrome *bd* oxidase, superoxide dismutase and hemerythrin-like proteins (French, Bell and Ward 2008; Forte *et al*. 2016; Berg *et al*. 2019). Other PSB were also found to oxidize thiosulfate and sulfide under aerobic chemolithoautotrophic conditions, using RubisCO and the Calvin cycle to assimilate CO_2_ (Kondratieva *et al*. 1976).

So far, there has not been much evidence of metabolic activity of GSB under aerobic conditions, even if Berg *et al*. (2019) observed that two species of GSB from Lake Cadagno possess genes encoding for terminal oxidases. This finding is corroborated by previous observations (Eisen *et al*. 2002; Frigaard *et al*. 2003; Ng *et al*. 2010) that GSB *Chlorobium tepidum* is equipped with several proteins against oxidative damage, suggesting that these bacteria can sustain aerobic conditions and that such enzymes play a role in protecting the cell when exposed to O_2_. GSB are considered strict anaerobes, thus sensitive to even low oxygen concentrations, and therefore can be expected to have limited capabilities for oxidative stress protection. Especially in presence of light, our results showed a clear reduction in the photosynthesis of *C. phaeobacteroides* 1VII D7 when exposed to 5% of oxygen (Fig. 3) with 3-fold less ^14^CO_2_ fixed compared to both other anaerobic conditions. Even in the dark, the presence of oxygen coincides with a lower chemo-oxidation value, different from that observed for PSB.

### Response to sulfide oxidation of PSB and GSB

In aquatic environments, productivity of anaerobic phototrophic sulfur bacteria normally correlates well to the amount of light reaching the anaerobic layers (Parkin and Brock 1980). A similar relationship can be determined between the amount of CO_2_ assimilated through anoxygenic photosynthesis by these organisms and the amount of light reaching the bacterial layer (Montesinos and van Gemerden 1986). Therefore, light is likely the main factor controlling the photosynthesis of anoxygenic phototrophic sulfur bacteria. Following these considerations, we investigated the rates of sulfide photo-oxidation of PSB *C. okenii* LaCa and *T. syntrophicum* Cad16^T^ and GSB *C. phaeobacteroides* 1VII D7.

The three strains had a similar response in the oxidation rates to variations in light intensity, albeit with considerable differences among species. All three strains, in fact, showed an increase in the sulfide uptake rate in direct proportion with the initial concentration of sulfide (Table 2). In general, however, the response to increasing light intensities is analogous to that recorded for GSB in the Chesapeake Bay (Luther, Ferdelman and Tsamakis 1988; Hanson *et al*. 2013; Findlay *et al*. 2015), where observations over the years confirmed light-dependent anaerobic sulfide uptake as a component of the sulfur cycle.

Overall, laboratory cultures of *C. okenii* LaCa showed higher *V_max_* values indicating a faster response to the presence of S^2−^ compared to both *C. phaeobacteroides* 1VII D7 and *T. syntrophicum* Cad16^T^, that anyway had lower *K* values, indicating their high sensitivity to light at low radiance (Table 2). GSB, in particular, can grow at the lowest light intensities of all known phototrophs (Manske *et al*. 2005; Haas *et al*. 2018). The strains used in this experiment were grown under laboratory conditions and exposed to light intensities much higher than those normally encountered in the chemocline of Lake Cadagno. Therefore, the higher light intensity irradiance necessary to obtain the saturation with laboratory grown cultures, compared to what observed in a previous work using fresh isolates (Danza 2018), might be due to a higher production of photoprotective pigments caused by the exposure to strong artificial light (Šlouf *et al*. 2012). Indeed, both dialysis bags PSB cultures showed, on average, a higher response to lower sulfide concentrations (V_max_) compared to laboratory cultures, particularly showing photo-oxidation activity even at 0.02 mM S^2^ (Fig. 4A and B). At the same sulfide concentrations, GSB *C. phaeobacteroides* had oxidation rates comparable to those observed in the laboratory cultures (Fig. 4C). The better response showed by dialysis bags cultures to lower sulfide concentrations is probably due to the 6-weeks incubation period in the chemocline of the lake during which the cultures acclimatized to the conditions of the natural environment, where they are exposed to a lower light irradiance but, at the same time, are more capable of using it. As shown above, the intensity of light used in the growth of the cultures is very important to regulate the affinity, especially of PSB, to small amounts of sulfide. As for the quality of light, we did not observed substantial differences in the growth rates using 3 different light spectra (data not shown).

As for CO_2_ assimilation, cell volume normalization of sulfide oxidation rates resulted in substantial differences, with large-celled *C. okenii* becoming by far the least efficient under any S^2−^ concentration (Figure S9, Supporting Information), suggesting that, for the purpose of this work, photosynthetic activity too should not be investigated in terms of cell volume, which tends to overestimate the real sulfide and light absorption of small-celled bacteria.

Of note, fitting the kinetic data of sulfide oxidation (Fig. 4) revealed a deviation from linearity in the dose/response curve (sulfide oxidation/light irradiance) of the three bacterial strains. All species displayed a kinetic curve with a Hill coefficient between 1.5 and 2.0 (data not shown). Hill coefficient was historically employed to quantify binding cooperativity to enzymes or transport proteins with multiple substrate binding sites (Hill 1910), and its usage further extended to quantify non-linear dose/response in different contexts (Ferrell and Machleder 1998). A typical example of binding cooperativity is the case of hemoglobin, which contains four oxygen binding sites, and a measured Hill coefficient of 2.8. This indicates a positive effect exerted by the binding of a oxygen molecule onto the next oxygen molecule binding (i.e. the *n*th oxygen bound to hemoglobin, facilitates the binding of the (*n*+1)th oxygen molecule). Similarly, in this present study, the deviation from the linear range observed in Fig. 4, indicates a homotypic form of cooperativity, in which the sulfide oxidation rate is enhanced at intermediate light irradiance in a switch-like manner. Further experiments are needed to determine if this effect is due to cooperative binding of sulfide molecules to the enzymes that metabolize them, due to enhanced import mechanisms on the cell surface after a dosage threshold, or at a more macroscopic level, at which cells might deploy a concerted response after a sulfide concentration is reached (e.g. by quorum sensing).

### Light-dependent intracellular variations

PSB *C. okenii* LaCa and *T. syntrophicum* Cad16^T^ displayed a significant alteration in the intracellular complexity in response to photo-oxidation of sulfide, as clearly shown by the SSC signal (Fig. 6). This increase in intracellular complexity is directly related to the formation of sulfur globules (S^0^) after the oxidation of sulfide. In the laboratory cultures, the increase in SSC signal observed after the incubation, and the relative maximal SSC values, positively correlated with the initial sulfide concentration (Fig. 6A–D), whereas in dialysis bags cultures a major increase in the internal complexity was observed at lower S^2−^ concentrations, particularly for *C. okenii* (Fig. 6E). The absence of sulfide oxidation and SSC variation recorded under dark incubation corroborates this observation (Figure S5b and c, Supporting Information). These findings are in line with those from Danza *et al*. (2017) and other studies (Casamayor *et al*. 2007; Frigaard and Dahl 2008) correlating sulfide oxidation with the intracellular accumulation of sulfur globules, as a result of their photosynthetic activity (Mas and Van Gemerden 1995). Moreover, the higher SSC values observed at low sulfide concentrations in dialysis bags seem to suggest that, under natural conditions, PSB reach the optimum at low sulfide concentrations. This consideration is corroborated by the fact that in the lake the lowest sulfide concentrations are found in the upper part of the bacterial layer, where light penetration is higher.

Anoxygenic phototrophic sulfur bacteria are also known for storing other inclusion bodies, such as glycogen, that might influence the SSC signal (Mas and Van Gemerden 1995). Moreover, it has been observed that accumulation of storage carbohydrates is usually coupled with the production of elemental sulfur during anoxygenic photosynthesis (Mas and Van Gemerden 1995; Casamayor *et al*. 2007). In PSB and GSB, the glycogen synthetic pathway involves ATP and α-D-glucose-1-phosphate as substrates of the enzyme glucose-1-phosphate adenyltransferase in the biosynthesis of ADPglucose, which is then transferred to an α-glucan primer to form the α-(1, 4) linkages in the polymer in a reaction catalized by glycogen synthase (Sirevåg 1995; Wilson *et al*. 2010; Machtey *et al*. 2012). The observed increase in ATP concentration in all the strains, particularly under the 0.4 and 1.2 mM S^2-^ concentrations, could then result from the achievement of conditions where electron transport initiated by light reaction triggers the production of the ATP and the reducing power needed for the synthesis and storage of glycogen. In fact, the synthesis of glycogen has been found to be maximal in presence of a high energy state of the cell (Preiss and Sivak 1999; Ballicora, Iglesias and Preiss 2004). The accumulation of glycogen, together with polyhydroxybutyrate, is supposed to be involved in the process of dark CO_2_ assimilation, particularly in *T. syntrophicum* Cad16^T^ (Storelli *et al*. 2014). Moreover, the little increases in SSC values of *C. phaeobacteroides* after the exposure to light (Fig. 6) were probably the consequence of variations in glycogen and other inclusion bodies detected by SSC, rather than a result of internal storage of S^0^, since it has been widely demonstrated that GSB secrete sulfur globules outside the cell (Frigaard *et al*. 2003; Frigaard and Dahl 2008; Holkenbrink *et al*. 2011).

Overall, the metabolic activity during the light-dependent sulfide oxidation did not affect the cell size (FSC signal, Figure S6, Supporting Information). Our data demonstrate that photosynthetic activity in phototrophic sulfur bacteria determined intracellular physiological alterations by the accumulation of inclusion bodies with no effects on cell size.

## CONCLUSIONS

In this paper, we highlight the ecological importance as primary producers of the anoxygenic phototrophic sulfur bacteria community living in the chemocline of Lake Cadagno. The ^14^CO_2_ assimilation rates of PSB *C. okenii* strain LaCa, PSB *T. syntrophicum* strain Cad16^T^ and GSB *C. phaeobacteroides* strain 1VII D7, corresponding to the 72.8% of the total community in Lake Cadagno, indicated that they are much more active in the presence of light via photosynthesis than in the dark via chemotrophy, both *in situ* and *in vitro*.

In both experiments, in the presence of light, small-celled PSB *T. syntrophicum* Cad16^T^ proved to be the most effective in CO_2_ photo-assimilation, followed by the other PSB *C. okenii* LaCa. As in previous studies, GSB *C. phaeobacteroides* 1VII D7 showed a low photo-assimilation activity also because of its very small size compared to PSB. Moreover, for all strains, the presence of oxygen strongly reduced the photosynthetic activity, while in absence of light, especially for PSB *C. okenii* LaCa, the presence of a small amount of O_2_ strongly increased the fixation of CO_2_. Interestingly, the condition of microaerophily (around 5% of O_2_) did not have the same amplifying effect for the other PSB *T. syntophicum* Cad16^T^, which suggests the presence of another different mechanism of chemotrophy. In general, dark CO_2_ fixation was substantially lower compared to phtosynthesis for all three species.

Photosynthetic CO_2_ fixation is directly correlated to the efficacy of pigments in collecting photons of light and to the capability of oxidizing reduced compounds, such as H_2_S. Here, we compared the ability of our three anoxygenic phototrophic sulfur bacteria strains to oxidize different concentrations of sulfide with increasing light intensities. As observed in the ^14^CO_2_ assimilation experiment, both PSB species were more efficient than *C. phaeobacteroides* 1VII D7 in terms of photosynthetic activity. Big-celled PSB *C. okenii* LaCa was able oxidize the highest amount of sulfide probably due to its large cell size. In fact, once the results were normalized to cellular biovolume, the small GSB *C. phaeobacteroides* 1VII D7 became the most efficient. After the 6-weeks incubation in Lake Cadagno chemocline inside dialysis bags, the cultures showed an higher affinity to lower sulfide concentration compared to laboratory cultures.

From an ecological point of view, at the level of individual species, *T. syntrophicum* Cad16^T^ seems to be the most effective in fixing CO_2_, especially in the presence of light, even if it is the least present in chemocline. Instead, the most abundant but also the smallest, *C. phaeobacteroides* 1VII D7, is theleast active in terms of CO_2_ fixation, besides being the most sensitive to oxygen. The largest and most efficient in the photo-oxidation of H_2_S *in vitro*, *C. okenii* LaCa, in our study does not not seem to be so strong in fixing CO_2_, as shown in the past. In the natural environment, however, it can produce a turbulence process known as bioconvection, which could represent a competitive advantage in the quest for the optimal environmental conditions and affect the effectiveness of other microorganisms.

## Supplementary Material

fiab010_Supplemental_File
