# Supplementary material for: Anoxygenic photo- and chemo-synthesis of phototrophic sulfur bacteria from an alpine meromictic lake
Source: FEMS Microbiol Ecol. 2021 Jan 29;97(3):fiab010. doi: 10.1093/femsec/fiab010 (PMC7947596; doi:10.1093/femsec/fiab010)
Supplement: fiab010_Supplemental_File [file fiab010_supplemental_file.docx]

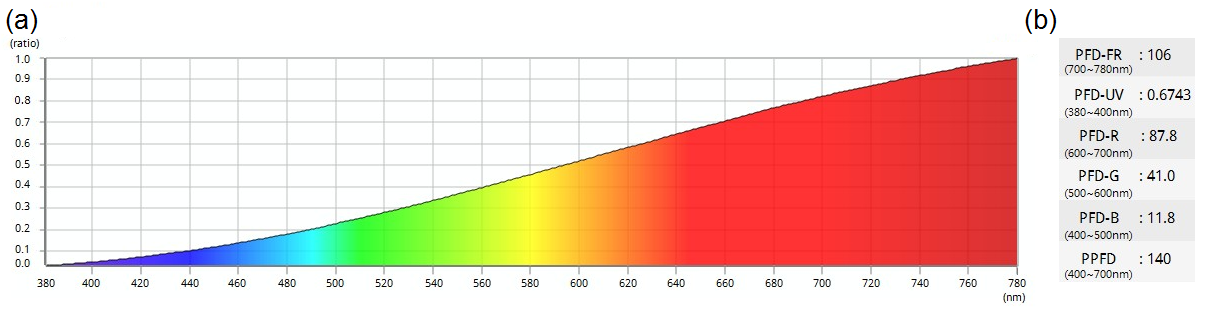


Figure S1 (a) Spectral power distribution and (b) different ranges of Photosynthetic Photon Flux Density (PFD) [μmol m^-2^ s^-1^] of the 100 W incandescent light bulb used to grow the bacterial cultures.


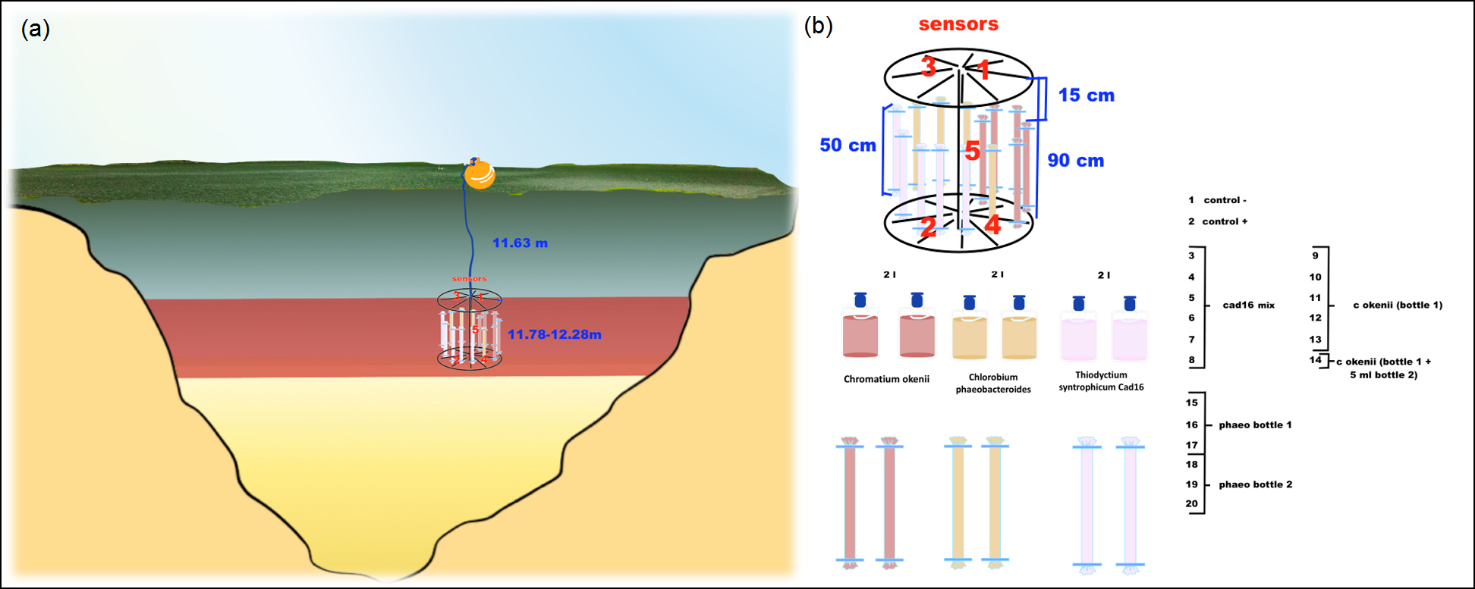


Figure S2 (a) Mooring scheme of the incubation experiment (initial depth, July to August) in Lake Cadagno and (b) set-up of the cultures in dialysis bags attached to a support grid. Red (b) and blue (a) numbers indicate the HOBO temperature and light sensors position and depth, respectively.

Figure S3 Relative light availability and temperature profiles (13 July –27 September 2019) of the HOBO logger in the middle of the rig at 11.8 m depth. Low light availability and temperature are characteristic for the depth of around 12.0 m. Recovery of the rig on the 22^nd^ August 2019 resulted in both a peak in temperature and light availability.


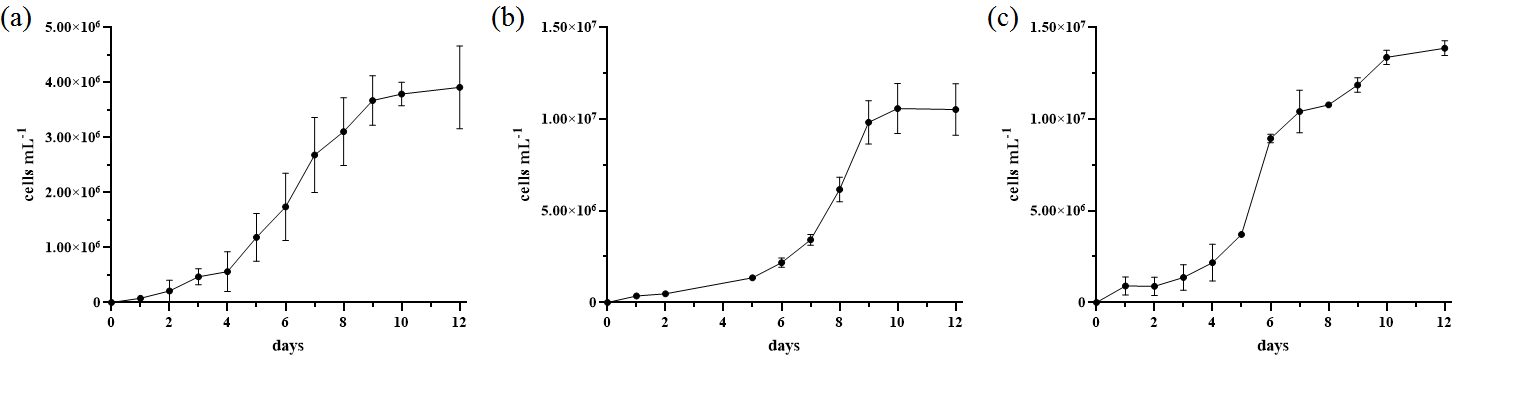


Figure S4 **Growth curves of anoxygenic photosynthetic sulfur bacteria.** (a) C. okenii, (b) T. syntrophicum and (c) C. phaeobacteroides. Error bars represent standard deviation (N = 3). If no error bars are shown, SD was smaller than the symbols used.


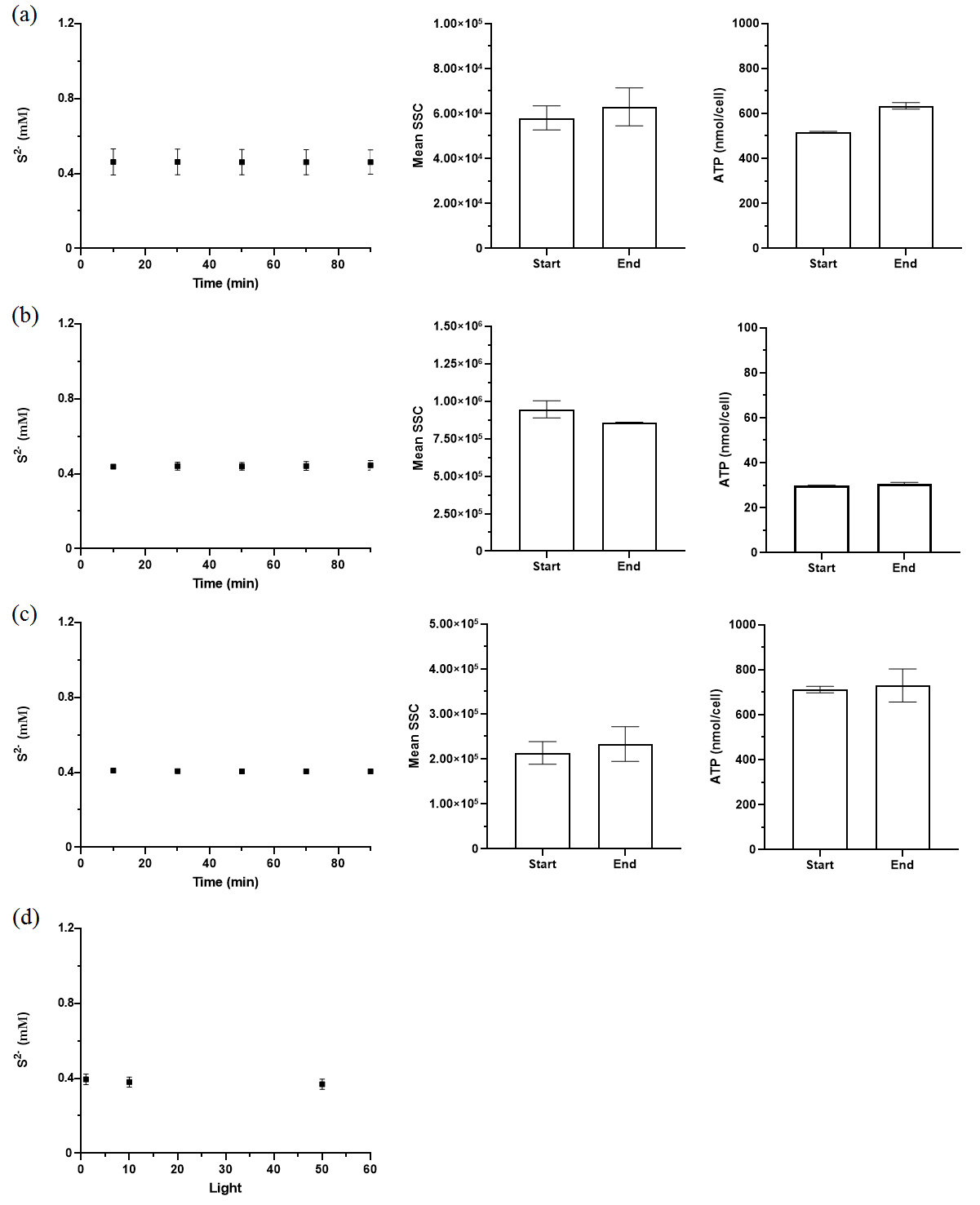


Figure S5 **Negative controls.** S^2-^ concentration [mM], mean SSC, ATP [nmol cell^-1^] for (a) C. phaeobacteroides, (b) T. syntrophicum and (c) C. okenii under dark incubation. (d) Culture medium with no cells under light incubation. Starting concentration was 0.4 mM. Error bars represent standard deviation (N = 3). If no error bars are shown, SD was smaller than the symbols used.


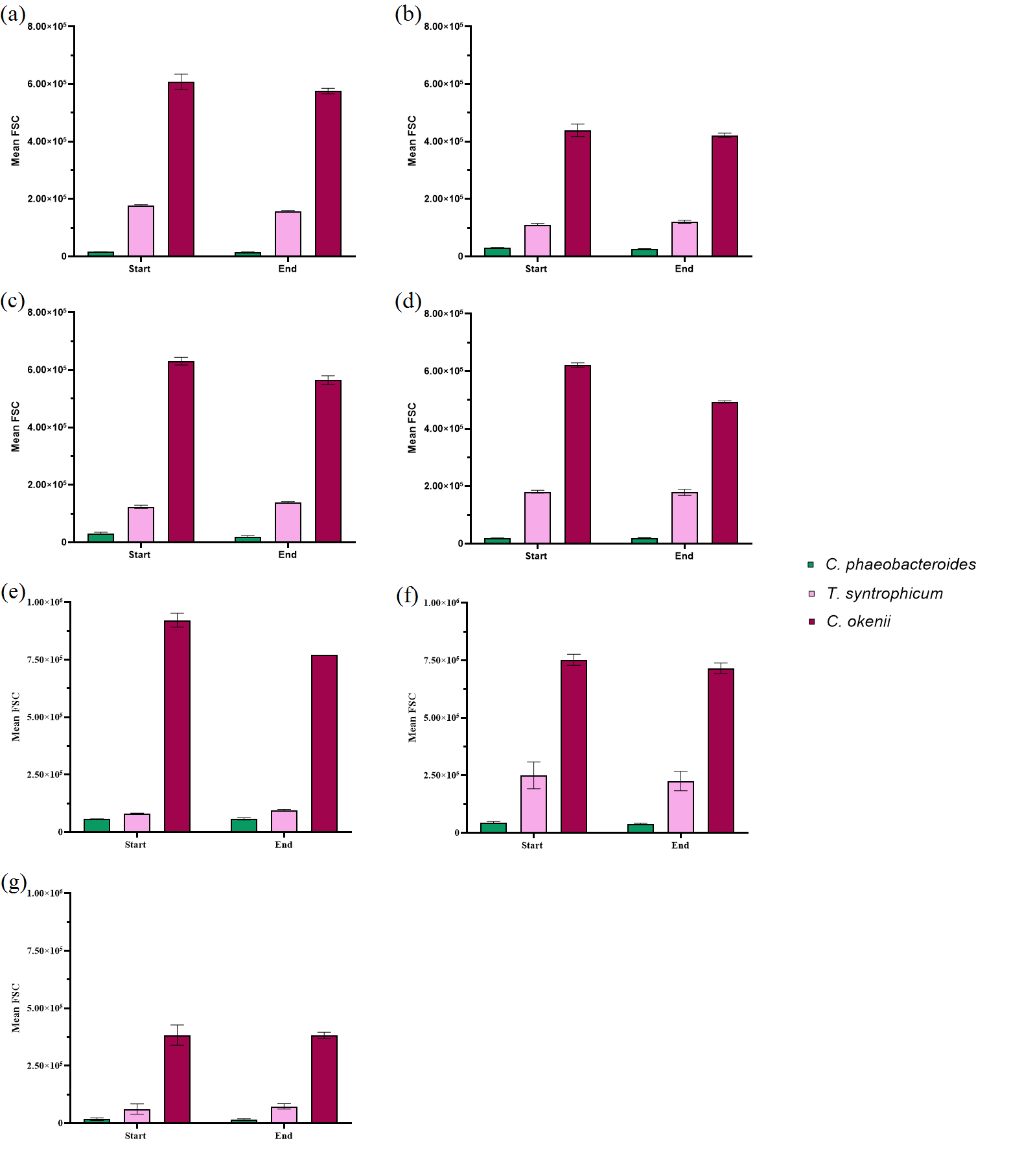


Figure S6 **Flow cytometry cellular dynamics in anoxygenic photosynthetic sulfur bacteria.** Mean FSC for laboratory cultures under (a) 0.02 mM S^2-^ (no H_2_S oxidation observed), (b) 0.04 mM S^2-^, (c) 0.4 mM S^2-^ and (d) 1.2 mM S^2-^ and for dialysis bags cultures under (e) 0.02 mM S^2-^, (f) 0.04 mM S^2-^ and (g) 0.4 mM S^2-^. Error bars represent standard deviation (N = 3). If no error bars are shown, SD was smaller than the symbols used.


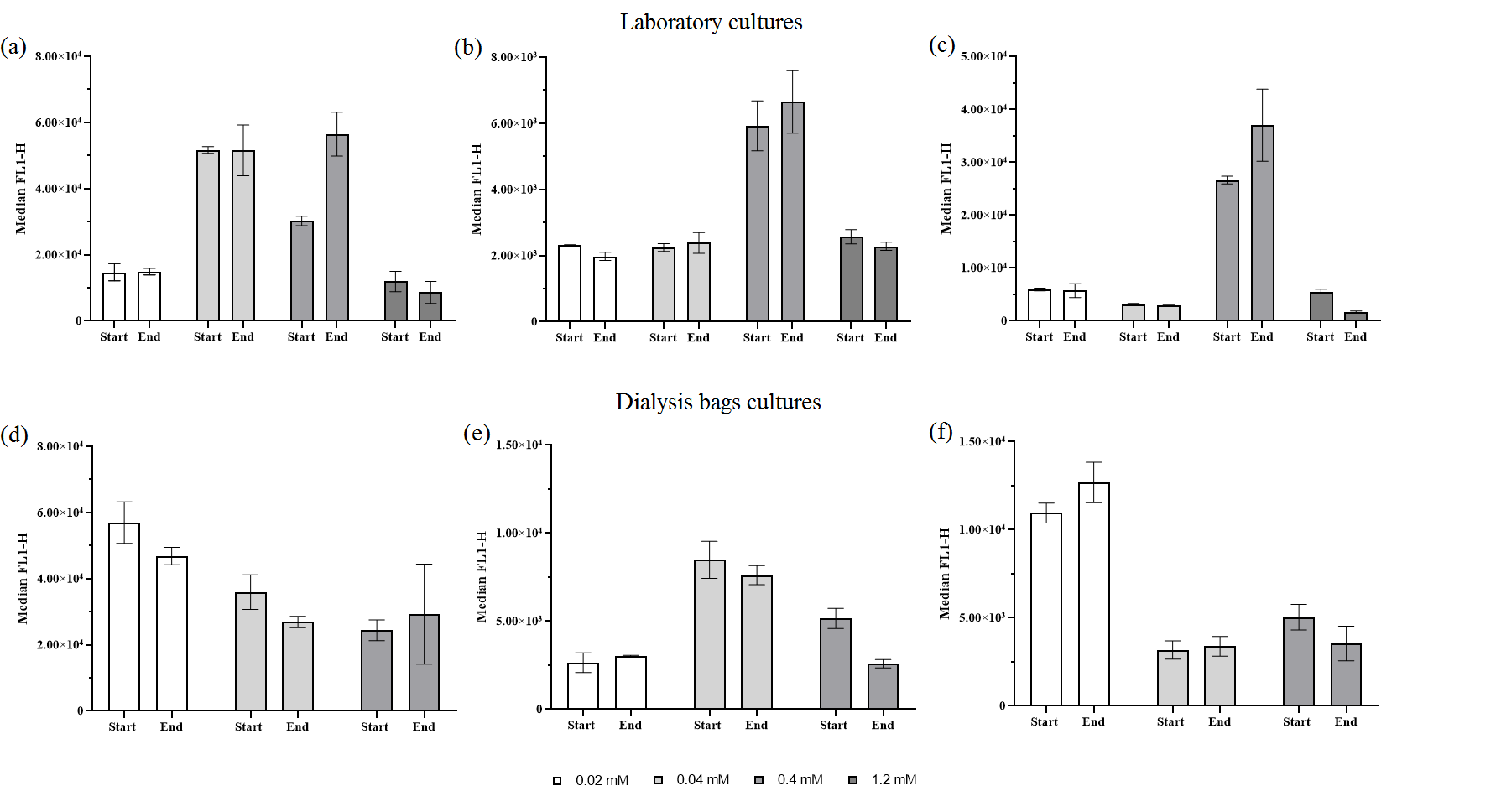


Figure S7 Nucleic-acid content [median FL1-H] for (a, d) C. okenii, (b, e) T. syntrophicum and (c, f) C. phaeobacteroides after SYBR Gold staining for relative detection of double or single stranded DNA or RNA. Error bars represent standard deviation (N = 3).


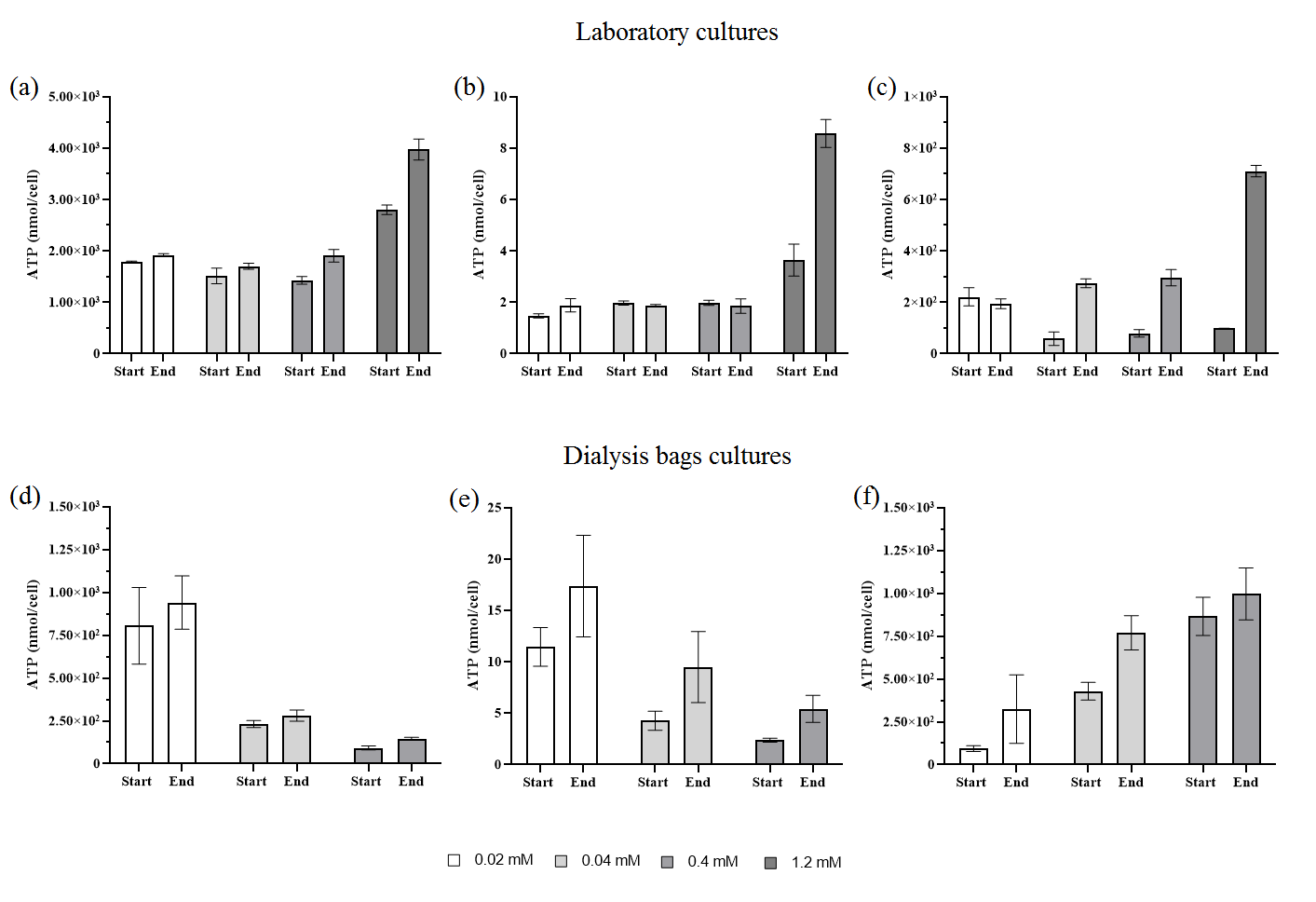


Figure S8 ATP concentrations [nmol cell^-1^] for (a, d) C. okenii, (b, e) T. syntrophicum and (c, f) C. phaeobacteroides. Error bars represent standard deviation (N = 3).


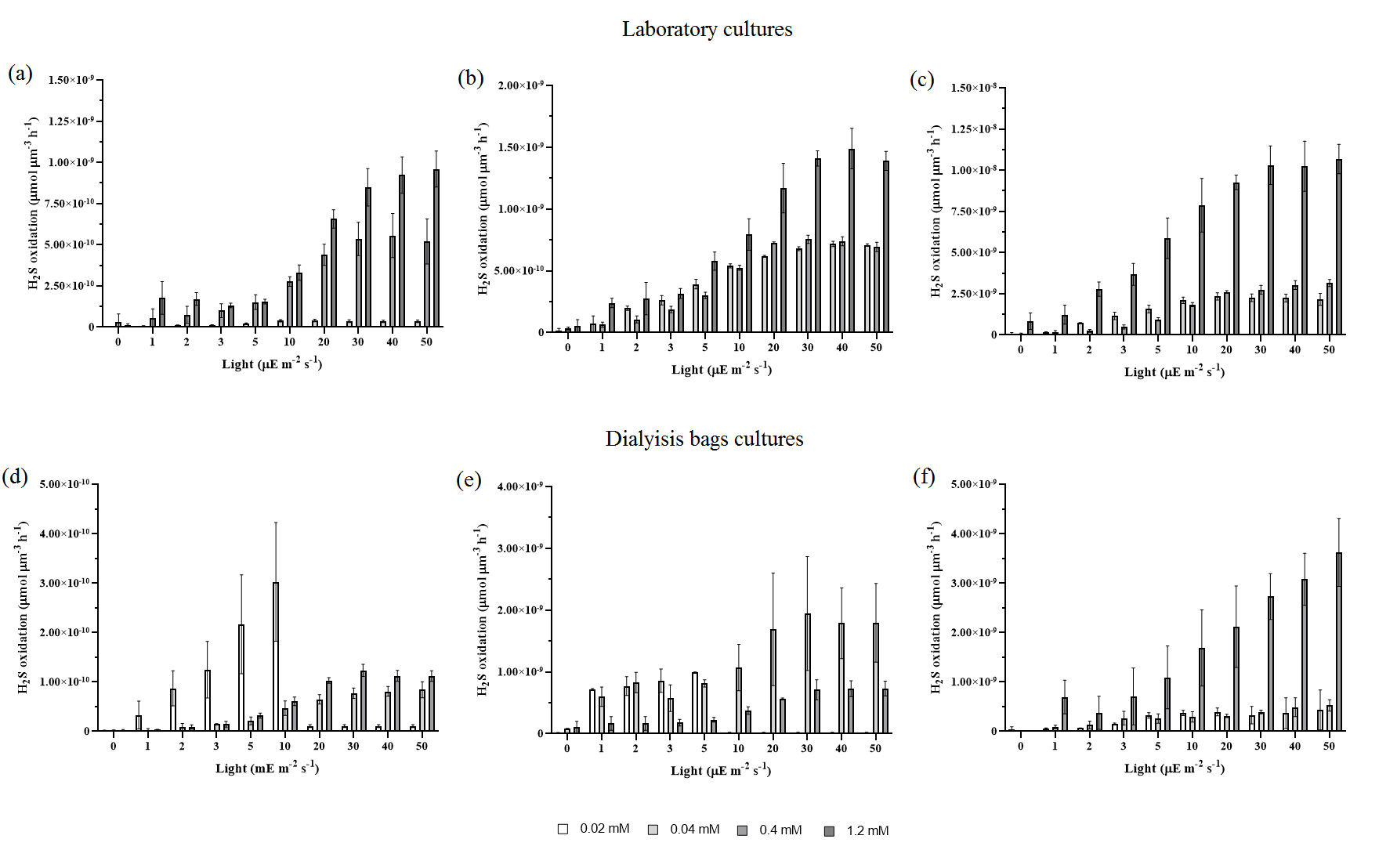


Figure S9 **Light-dependent sulfide oxidation rates normalized to cell biovolumes**. H_2_S oxidation rate [μmol μm^-3^ h^-1^] vs light [μmol m^-2^ s^-1^] for (a, d) C. okenii, (b, e) T. syntrophicum and (c, f) C. phaeobacteroides. Error bars represent standard deviation (N = 3). If no error bars are shown, SD was smaller than the symbols used.
